# The zymogenic form of SARS-CoV-2 main protease: A discrete target for drug discovery

**DOI:** 10.1016/j.jbc.2024.108079

**Published:** 2024-12-14

**Authors:** Pavel Novotný, Jana Humpolíčková, Veronika Nováková, Stancho Stanchev, Kvido Stříšovský, Michala Zgarbová, Jan Weber, Robin Kryštůfek, Jana Starková, Martin Hradilek, Adéla Moravcová, Jana Günterová, Kathrin Bach, Pavel Majer, Jan Konvalinka, Taťána Majerová

**Affiliations:** 1 Institute of Organic Chemistry and Biochemistry of the Czech Academy of Sciences, Prague, Czech Republic; 2 Faculty of Science, Department of Physical and Macromolecular Chemistry, Charles University in Prague, Prague, Czech Republic; 3 Faculty of Science, Department of Genetics and Microbiology, Charles University in Prague, Prague, Czech Republic; 4 Department of Biochemistry and Microbiology, University of Chemistry and Technology Prague, Prague, Czech Republic; 5 Faculty of Science, Department of Biochemistry, Charles University in Prague, Prague, Czech Republic

**Keywords:** virus, SARS-CoV-2 main protease, nsp5, precursor, autoprocessing, maturation, inhibitor, activation, cell-based assay, Förster resonance energy transfer (FRET), fluorescence cross-correlation spectroscopy (FCCS), fluorescence life-time imaging, protease

## Abstract

Severe acute respiratory syndrome coronavirus 2 (SARS-CoV-2) main protease (M^pro^) autocatalytically releases itself out of the viral polyprotein to form a fully active mature dimer in a manner that is not fully understood. Here, we introduce several tools to help elucidate differences between *cis* (intramolecular) and *trans* (intermolecular) proteolytic processing and to evaluate inhibition of precursor M^pro^. We found that many mutations at the P1 position of the N-terminal autoprocessing site do not block *cis* autoprocessing but do inhibit *trans* processing. Notably, substituting the WT glutamine at the P1 position with isoleucine retains M^pro^ in an unprocessed precursor form that can be purified and further studied. We also developed a cell-based reporter assay suitable for compound library screening and evaluation in HEK293T cells. This assay can detect both overall M^pro^ inhibition and the fraction of uncleaved precursor form of M^pro^ through separable fluorescent signals. We observed that inhibitory compounds preferentially block mature M^pro^. Bofutrelvir and a novel compound designed in-house showed the lowest selectivity between precursor and mature M^pro^, indicating that inhibition of both forms may be possible. Additionally, we observed positive modulation of precursor activity at low concentrations of inhibitors. Our findings help expand understanding of the SARS-CoV-2 viral life cycle and may facilitate development of strategies to target precursor form of M^pro^ for inhibition or premature activation of M^pro^.

Severe acute respiratory syndrome coronavirus 2 (SARS-CoV-2) is a complex, positive-sense RNA virus with several potential drug targets (1). Following viral entry into a cell, viral genomic RNA is released and translated by the host machinery, producing polyproteins pp1a and pp1ab. Polyprotein pp1ab is an extended version of pp1a that arises from a ribosomal frame shift at a specific conserved RNA structure. This slippery sequence ensures the production of pp1a and pp1ab in a fixed stoichiometry (2, 3). The two polyproteins are subsequently cleaved into 16 nonstructural proteins (nsps). Most cleavages are performed by the homodimeric main protease (M^pro^) (E.C. 3.4.22.69), referred to as nsp5, M^pro^, or chymotrypsin-like 3C protease; one M^pro^ monomer is embedded in each polyprotein (4). M^pro^ releases transmembrane proteins nsp4 and nsp6 (5), which are involved in the formation of double membrane vesicles (DMVs) (6, 7). DMVs are endoplasmic reticulum (ER) - derived replication organelles that support the spatial organization of the replication complex and protect nascent viral RNA against host defenses (6). The order in which nsps are released from pp1a and pp1ab depends on the primary sequence of the processing sites (4, 8, 9, 10, 11, 12) and their steric accessibility (9, 10, 11).

Different rates of processing individual nsps affect the appropriate timing of steps in the viral replication cycle. For example, M^pro^ (nsp5) is activated by removal of the N-terminal sequence of its zymogen, whereas M^pro^ with C-terminal extensions retains normal activity (13, 14). Cleavage of nsp4/nsp5 is rapid (4, 8, 12) and essential not only for M^pro^ activation but also for the release of DMV-forming nsp4 (15). In comparison, cleavage of nsp5/nsp6 is slower (4, 8, 12). This slower processing may result in transient M^pro^ anchoring *via* fusion with nsp6 to the ER membrane (perhaps prior to DMV formation) without compromising enzyme activity. Similarly, nsp10 is promptly cleaved out of pp1ab (4, 8, 9, 10, 11, 12). This scaffolding protein and activator of viral RNA capping enzymes interacts with the more slowly released nsp14 (16, 17, 18, 19).

M^pro^ also cleaves host proteins to promote optimal viral replication (20, 21, 22, 23, 24, 25). Some of these cleavages are responsible for the cytotoxicity of M^pro^ (26, 27, 28). Research on proteases of other positive-sense RNA viruses has highlighted the essential role of correctly timed proteolysis during the viral replication cycle (29, 30, 31, 32, 33, 34, 35, 36, 37, 38, 39, 40, 41, 42, 43). In some cases, partially cleaved intermediate fusion proteins may play a specific role (44, 45, 46).

Due to its crucial role during polyprotein processing, M^pro^ is considered an important drug target (47, 48). M^pro^ is a homodimeric cysteine proteinase (49, 50). Each protomer (nsp5), harboring one active site, is part of one polyprotein. Nsp5 is embedded in an extramembrane loop on the cytosolic site of the ER between the transmembrane proteins nsp4 and nsp6 (51) (Fig. 1*A*). Maturation of M^pro^ involves dimerization and processing, although the order of these two steps remains unclear (13, 52, 53, 54, 55).

**Figure 1:**
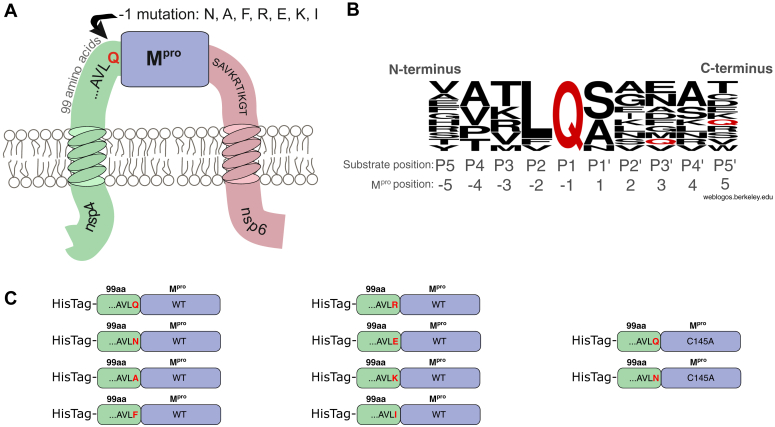
**M**^**pro**^**substrate specificity and location of preM**^**pro**^**on the ER membrane**. *A*, M^pro^ is located between nonstructural protein 4 on the N terminus and nonstructural protein 6 on the C terminus. *B*, comparison of sequences from M^pro^ cleavage sites occurring in viral polyproteins using WebLogo 2.8.2 (62). M^pro^ strongly prefers glutamine (*red*) in the P1 position. *C*, precursor variants engineered from the anticipated 99 extramembrane amino acids of nsp4 and M^pro^ itself. The last C-terminal residue of nsp4, numbered −1 in relation to M^pro^, was mutated to create variants with different self-cleavage properties. ER, endoplasmic reticulum; preM^pro^, precursor form of M^pro^; M^pro^, main protease.

The monomeric precursor form of M^pro^ likely comprises a dynamic ensemble of conformers. A minority of these molecules may have a similar conformation as mature M^pro^ (56) and may form transient dimers (57, 58). Experiments involving dimerization-defective precursors of M^pro^ have shown that the precursor is characterized by deficient enzyme activity and that N-terminal cleavage, which is performed earlier than C-terminal cleavage, can occur in an intramolecular manner (*i*.*e*., *in cis*) (59). Processing of the N-terminal intradimeric maturation site, followed by C-terminal interdimeric cleavage, was illustrated by crystallographic “snapshots” of the C145S mutant of M^pro^. Slower processing of this mutant allowed crystallization at various stages of maturation (13). Studies of SARS-CoV-1 have demonstrated interdimeric cleavage *in trans* and the occurrence of N-terminal cleavage of M^pro^ prior to C-terminal processing (60, 61).

All natural M^pro^ cleavage sites contain a conserved glutamine in the P1 position (Fig. 1*B*, created with WebLogo (62)), while other positions vary. Hydrophobic residues and small uncharged residues are typically preferred in the P2 and P1′ positions, respectively. Studies of substrate–enzyme interactions are helpful for drug design (63) and have aided development of several drugs targeting SARS-CoV-2 M^pro^. The U.S. Food and Drug Administration–approved coronavirus disease 2019 treatment Paxlovid contains nirmatrelvir, a covalent reversible inhibitor of M^pro^, and the pharmacokinetic booster ritonavir (https://www.fda.gov/news-events/press-announcements/fda-approves-first-oral-antiviral-treatment-covid-19-adults). A similar combination therapy involving the protease inhibitor simnotrelvir and ritonavir is sold in China under the brand name Xiannuoxin (64). A noncovalent protease inhibitor, ensitrelvir (sold under the brand name Xocova), is approved in Japan (65).

Targeting viral proteases in their zymogenic precursor forms could block the initial irreversible step in the “domino cascade” of protease activation (66, 67, 68). Accumulation of uncleaved precursors could impair production of viable viral progeny *via trans-*dominant inhibitory effects (67, 68, 69, 70, 71). In addition to inhibition, aberrantly timed or excessive activation of viral protease can be fatal to the virus, as demonstrated for HIV (34, 69, 72, 73, 74, 75, 76, 77). Although the design of activators seems challenging, some examples have been described in the literature (78, 79, 80), including one clinically used small molecule (81).

Here, we mutated the P1 position (Q-1) of the N-terminal cleavage site of precursor M^pro^ to suppress autocleavage at this site. We found that substrate specificity for *cis* autocleavage (intramolecular) is broader than for cleavage *in trans* (intermolecular), but we were able to purify intact precursor M^pro^. For cell-based studies, we developed an assay inspired by our previous work on HIV protease (67, 82, 83). We fused mCherry to M^pro^ flanked by 10 amino acids on each side, followed by C-terminal eGFP. We used two readouts for the encoded protease activity: FRET between eGFP and mCherry (67, 84, 85) in the uncleaved reporter and low expression levels of the reporter in the presence of active M^pro^ (26). When M^pro^ inhibition results in accumulation of uncleaved reporter, the FRET signal of the EGFP-mCherry pair can be detected. Upon inhibition of only the released mature protease, fluorescence of the isolated fluorophores can be observed, as the activity of precursor M^pro^ allows proteolysis of the reporter and consequent spatial separation of mCherry and eGFP.

We used biochemical tools to test small molecules as potential modulators of precursor activity. Initially, we screened a set of commercial and newly designed compounds using our fluorescent cell-based assay. We included the commercially available compounds boceprevir, GC376 (86), and bofutrelvir (87), as well as clinically used ensitrelvir (88) and nirmatrelvir (89). We designed three compounds: nonpeptide compound 1 is an indole trifluoromethylpyridinyl ester (derived from an indole chloropyridinyl ester (90)). Compound 2 is derived from GC376 and harbors glutamine in the P1 position instead of the original γ-lactam surrogate. Compound 3 is a peptide derivative with an α-ketoamide warhead, which enables extension of inhibitors to the Px’ positions (49, 91). The best compounds from this screen were studied in more detail in infected Calu-3 cells and in an assay with recombinant enzymes. All the compounds preferentially inhibited mature M^pro^. Compound 3 and bofutrelvir exhibited the smallest differences between inhibition of the precursor and the mature form. At low inhibitor concentrations, we observed positive modulation of activity of the recombinantly prepared precursor. Interestingly, compound 3 showed an initial phase of activation followed by a slow onset of inhibition of precursor M^pro^.

## Results

### Construction of precursor variants for expression in *E*. *coli*

To generate an M^pro^ precursor form that does not undergo maturation during proteosynthesis and purification, we constructed plasmids for expression in *Escherichia coli*. The resulting recombinant fusion proteins comprise the last 99 amino acids of nsp4 linked to the full-length M^pro^ sequence (Fig. 1, *A*–*C*). The conserved glutamine in the −1 position, relative to the N terminus of M^pro^ (Fig. 1*B*), was replaced by amino acids with different physicochemical properties (Fig. 1*C*). For reference, we also introduced the C145A mutation into the active site of several precursor variants. All the variants bear an N-terminal decahistidine tag (His Tag).

### N-terminal processing is not blocked by some amino acid replacements in the conserved P1 position of the cleavage site

**Figure 2:**
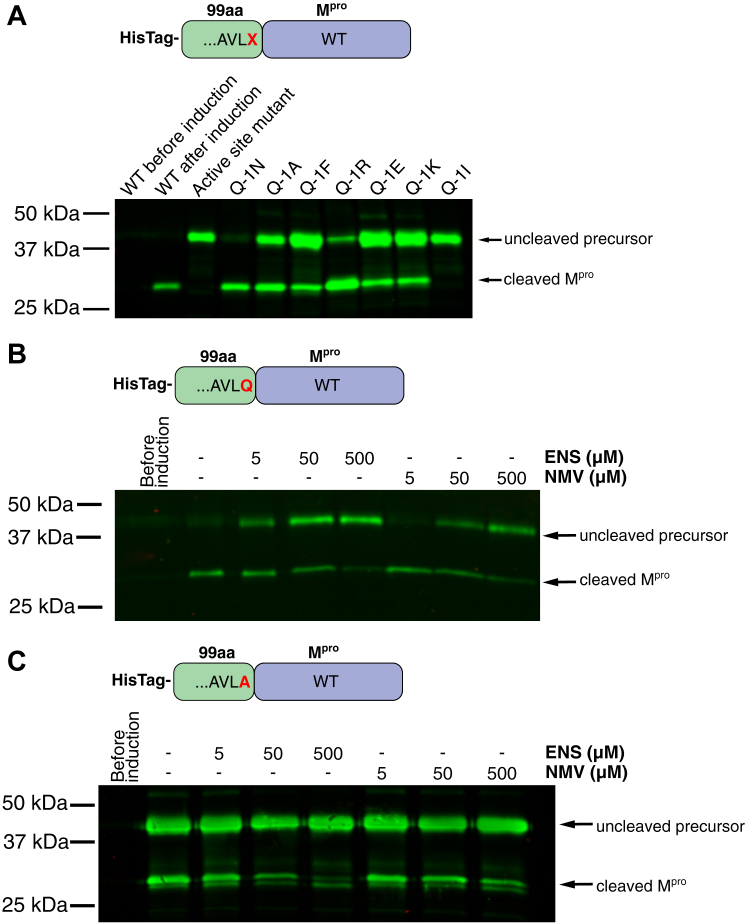
**Cleavage of recombinant precursor M**^**pro**^**variants during expression in *Escherichia coli***. *A*, schematic representation of the model preM^pro^. Position X was occupied by glutamine (the WT variant), asparagine, alanine, phenylalanine, arginine, glutamic acid, lysine, or isoleucine. The first lane contains sample transformed with WT preM^pro^ protease before induction by IPTG. Subsequent lanes contain the following samples after the induction by IPTG: WT preM^pro^, active site mutant (C145A), asparagine mutant (Q-1N), alanine mutant (Q-1A), phenylalanine mutant (Q-1F), arginine mutant (Q-1R), glutamic acid mutant (Q-1E), lysine mutant (Q-1K), and isoleucine mutant (Q-1I). *B*, expression of active WT protease or (*C*) the Q-1A mutant in the presence of the inhibitors nirmatrelvir (NMV) and ensitrelvir (ENS). preM^pro^, precursor form of M^pro^; M^pro^, main protease.

Most of the P1 (Q-1) mutations did not entirely block proteolytic processing (Fig. 2*A*). The only precursor variant with no observable cleavage had isoleucine in the Q-1 position (Q-1I). This variant, hereafter referred to as preM^pro^, was used in subsequent experiments as a model M^pro^ precursor.

The Q-1A mutant, purified using the nickel^2+^ ion that has been coupled to nitrilotriacetic acid (Ni-NTA) affinity purification *via* N-terminal HisTag, followed by size-exclusion chromatography, was used to confirm the authenticity of the released M^pro^ N terminus. Slow processing of the Q-1A precursor continued even after Ni-NTA purification. Western blot and subsequent N-terminal sequencing based on Edman degradation confirmed the authentic N terminus of M^pro^ (Ser-Gly-Phe-Arg), identical to that of WT M^pro^.

Processing of the WT model precursor M^pro^ (Fig. 2*B*) and the Q-1A mutant (Fig. 2*C*) can be blocked by M^pro^-specific inhibitors in a dose-dependent manner, indicating that N-terminal processing is mediated by the activity of the coronaviral protease.

### Canonical glutamine in the P1 position is required for *in trans* cleavage by active mature or precursor M^pro^

**Figure 3:**
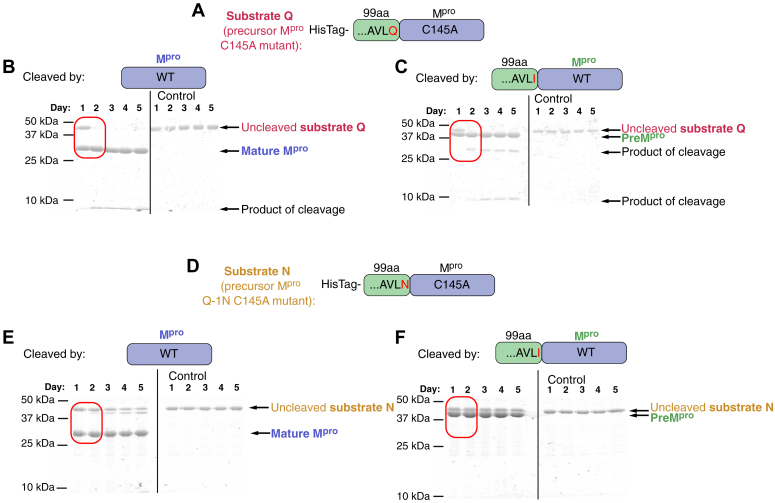
**SDS-PAGE analysis of cleavage of precursor M**^**pro**^**active site mutants (acting as substrates) by mature or precursor M**^**pro**^. After 5 days of cleavage at room temperature, products were analyzed by SDS-PAGE and stained by Coomassie Brilliant Blue G250. *A*, scheme of the active site mutant of preM^pro^ acting as a substrate Q, with glutamine retained in the P1 position. The active site cysteine was mutated to alanine (C145A), rendering this preM^pro^ incapable of autocatalytic cleavage. The histidine tag on the N terminus was used for purification and was not cleaved out of substrate Q, enabling the substrate to be distinguished from active precursor protease on gels. *B* and *C*, cleavage of substrate Q in *trans* (intermolecularly) by mature M^pro^ (B) or model active preM^pro^ with a Q-1I mutation (C). In each panel, samples on the *left* in lanes 1 to 5 contain a mixture of two proteins taken on day one (*lane 1*) and day five (*lane 5*). Lanes 1 to 5 on the *right* contain only substrate Q as a negative control. *D*, scheme of preM^pro^ used as substrate N. This variant contains Q-1N and C145A mutations and an N-terminal His-tag. *E* and *F*, active mature M^pro^ (*E*) or precursor M^pro^ (*F*) was mixed with substrate N and cleavage was monitored over 5 days (lanes 1–5 on the *left*). Lanes 1 to five on the *right* in each panel contain only substrate N as a negative control. When glutamine is in the P1 position, the substrate is cleaved (substrate Q, *red rectangles* in *panels B and C*). When asparagine is in the P1 position, no cleavage is observed (substrate N, *red rectangles* in *panels E and F*). preM^pro^, precursor form of M^pro^; M^pro^, main protease.

To evaluate substrate sequence requirements for *in cis* autocleavage of precursor M^pro^ and *in trans* cleavage of the precursor by the mature active dimer, we performed a series of experiments with recombinant proteins. First, we mixed the active site mutant precursor M^pro^(C145A) (named substrate Q, Figs. 3*A* and S1*A*) with mature M^pro^ or preM^pro^. Cleavage was monitored over the course of 5 days by Coomassie-stained SDS-PAGE. Notably, cleavage of substrate Q was observable only after addition of mature M^pro^ or preM^pro^ (Fig. 3, *B* and *C*). Cleavage *in trans* was prevented by adding the M^pro^ specific inhibitors ensitrelvir and nirmatrelvir (Fig. S1, *B–C*). This setup acts as a positive control where the natural cleavage site of the protein substrate Q is cleaved by mature M^pro^ or preM^pro^ *in trans* (intermolecularly).

We next investigated the Q-1N active site mutant precursor M^pro^ as another possible model substrate due to the high similarity between asparagine and glutamine (precursor M^pro^(Q-1N C145A), named substrate N (Figs. 3*D* and S1*D*),). We detected no cleavage of substrate N by M^pro^ or preM^pro^ over a period of 5 days, with daily analysis by SDS–PAGE (Figs. 3, *E* and *F*, S1, *E*–*F*). Hence, in contrast to the broader substrate specificity detected for *in cis* autocleavage (Figs. 2*A* and 3*C*), *in trans* cleavage is observed only with the canonical glutamine in the P1 position (Figs. 3, *A*–*F*, S1, *A*–*F*).

To support our results obtained with protein substrates (Fig. 3, *A*–*F* and S1, *A*–*F*), we engineered dodecapeptides derived from the N-terminal M^pro^ cleavage site with amino acid replacements in the P1 position. Only the peptide with the WT sequence was cleaved by M^pro^ and preM^pro^, while peptides with mutations of Q-1 to A or N remained intact (Fig. S2), in agreement with experiments using protein substrates. These results confirm the different substrate sequence requirements for the *cis* and *trans* cleavages.

### Construction of a eukaryotic cell-based reporter system to assess M^pro^ inhibition and impaired precursor processing by small molecules

**Figure 4:**
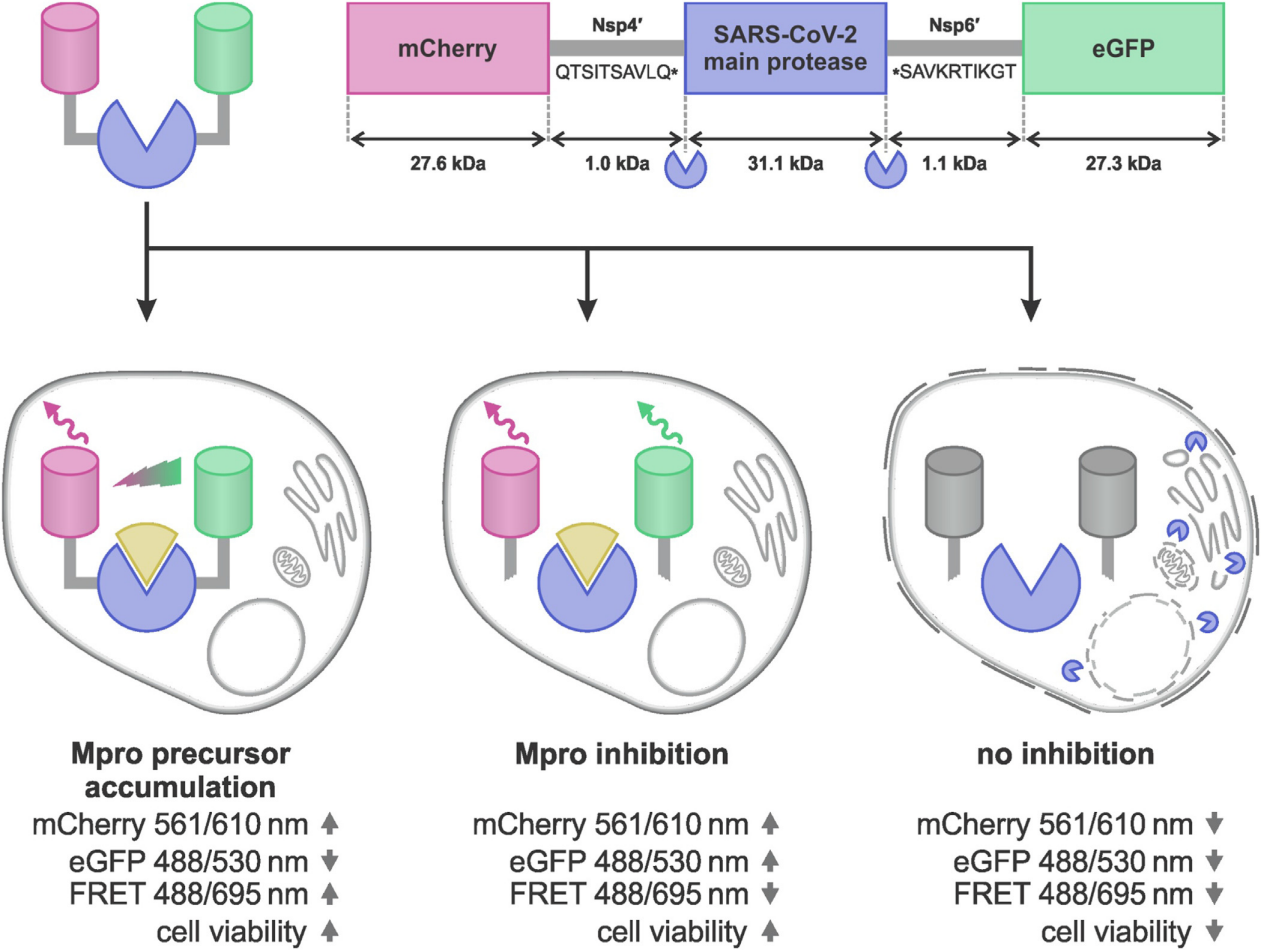
**Scheme of the reporter used in the cell-based assay.** M^pro^ flanked by 10 amino acids from the natural polyproteins on both termini (repM^pro^, *blue*) was fused with mCherry (*red*) at the N terminus and eGFP (*green*) on the C terminus to yield the reporter mCherry-repM^pro^-eGFP. When expressed in HEK293T cells, three possible scenarios can occur. First, M^pro^ is blocked in its precursor form and a FRET signal is detected in an inhibitor-dose–dependent manner. Second, M^pro^ is released from the precursor and inhibited in its mature form; the FRET signal disappears. Signals from the isolated fluorophores can be observed in an inhibitor-dose–dependent manner. Third, active M^pro^ lowers all the fluorescent signals. M^pro^, main protease.

To complement our work with recombinant preM^pro^, we developed a cell-based assay to study the precursor in a eukaryotic cell system. M^pro^, flanked on each side by 10 adjacent amino acids corresponding to the C terminus of nsp4 and the N terminus of nsp6 (repM^pro^), was inserted between the fluorescent proteins mCherry and eGFP. In this artificial precursor, the fluorescent proteins are sufficiently close to enable FRET (Fig. 4). The FRET signal is proportional to the amount of the precursor form. When the protease is first released from the reporter and then inhibited in its mature form, mCherry and eGFP are released, and the FRET signal disappears. However, it is still possible to measure the fluorescence of isolated mCherry and eGFP. We further exploited the fact that M^pro^ is cytotoxic when expressed in cells, reflected in decreased production of the reporter. Thus, in the absence of an M^pro^-specific inhibitor, the fluorescent signals of mCherry and eGFP are low, and they rise upon its inhibition. We also cloned a control reporter incorporating M^pro^ (C145A). Fig. 4 shows a schematic representation of these reporters.

**Figure 5:**
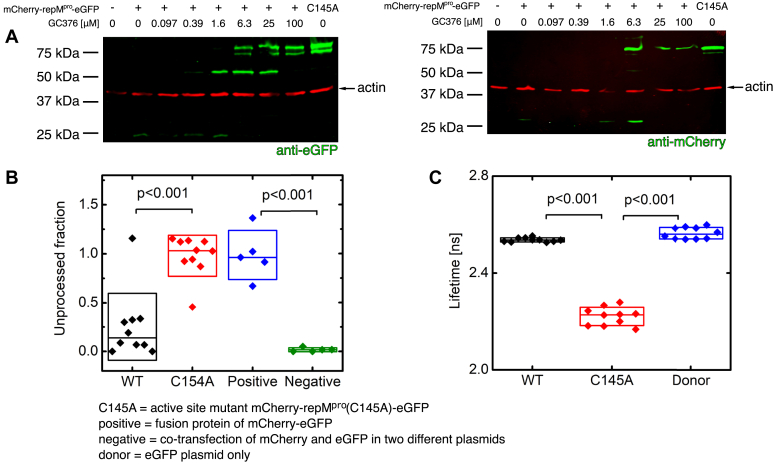
**Immunoblots from HEK293T cell lysates and microscopic studies of the M**^**pro**^**cell reporter**. *A*, HEK293T cells transfected by the reporter in the presence of increasing concentrations of the inhibitor GC376, visualized by (*left*) anti-eGFP antibody or (*right*) anti-mCherry antibody. The order of samples is the same in both immunoblots. C145A stands for the negative control mCherry-repM^pro^(C145A)-eGFP reporter with M^pro^-inactivating mutation. The unprocessed fraction was quantified by Image Studio Lite (Li-Cor). Precursor-to-actin signal ratios were as follows for the last four lanes: 0.45, 0.61, 0.64 and 2.23 (for eGFP blot) and 5.97, 3.10, 5.66, and 7.86 (for mCherry blot). *B*, characterization of the reporter by dual-color fluorescence cross-correlation spectroscopy (FCCS). Relative correlation of the fluorescence signals of mCherry and eGFP due to joint motion was low in cells expressing mCherry-repM^pro^-EGFP (due to autocatalytic release of mCherry and eGFP) and high in cells expressing the inactive mutant mCherry-repM^pro^C145A-eGFP (indicating retained fusion between mCherry and eGFP). As a positive control, a fusion protein of eGFP and mCherry lacking M^pro^ (mCherry-eGFP) was expressed, and as a negative control, eGFP and mCherry were coexpressed from separate cotransfected plasmids (pmGFP-N1 and pmCherry-C1). Paired Students *t* test was used for statistical evaluation of at least pentaplicates. *C*, characterization of the reporter by fluorescence lifetime imaging microscopy (FLIM). The graph shows the obtained fluorescence lifetimes of the donor (eGFP) in three conditions: in cells expressing mCherry-repM^pro^-eGFP (the lifetime is not shortened by energy transfer, because eGFP is released from the fusion by M^pro^ and the short range interaction between eGFP and mCherry vanishes), in cells expressing mCherry-repM^pro^ C145-EGFP (the lifetime is shortened due to energy transfer between eGFP and mCherry, which are brought close together in the uncleaved fusion protein), and in cells transfected only by a plasmid expressing free eGFP (control, the lifetime is not shortened due to lack of an interaction partner). Examples of FLIM cell images are in Fig. S3. Paired Student *t* test was used for statistical evaluation of decaplicates.

Autocleavage of the reporter was confirmed by SDS–PAGE of cell lysates, followed by immunoblotting and detection by anti-eGFP and anti-mCherry antibodies (Fig. 5*A*). The variant bearing WT active protease had a low or undetectable signal on the immunoblot in the absence of the M^pro^-specific inhibitor GC376. Increasing the concentration of GC376 led to the appearance of two detectable bands, and higher concentrations blocked reporter processing altogether. The variant containing M^pro^ (C145A) behaved similarly to the WT reporter treated with a high concentration of GC376; only the unprocessed fusion protein was detectable. This supports utility of the C145A mutant as a control resembling full protease inhibition.

We next studied the processing of the reporter using fluorescence cross-correlation spectroscopy (FCCS) (Fig. 5*B*), a single-molecule method that allowed us to evaluate dynamic movements of the mCherry and eGFP fluorophores. The cross-correlation between their respective red and green fluctuating signals enabled us to assess the relative fraction of unprocessed reporter. A low cross-correlation was observed with the WT reporter, indicating that mCherry and eGFP moved independently, which corresponds to cleavage of the artificial mCherry-repM^pro^-eGFP fusion protein and the release of both fluorophores. The same pattern was observed when cells were cotransfected with two plasmids encoding the isolated fluorescent proteins eGFP and mCherry. The cross-correlation remained high for mCherry-repM^pro^ (C145A)-eGFP, confirming that both the fluorescent molecules remain fused. Similar behavior was observed when a simple mCherry-eGFP fusion was used as a positive control (Fig. 5*B*).

Next, we evaluated the expression and autocleavage of mCherry-repM^pro^-eGFP using FRET combined with fluorescence lifetime imaging (FLIM), a single-molecule microscopic technique focusing on the spectroscopic properties of the donor (eGFP in this case, Fig. 5*C*). The fluorescence lifetime of eGFP is shortened by energy transfer when it is in sufficient proximity to a suitable acceptor fluorophore (mCherry). We compared the fluorescence lifetime of eGFP in cells expressing the reporter bearing WT M^pro^ or the C145A mutant, as well as in cells expressing eGFP only (Figs. 5*C* and S3). Cells expressing the WT reporter showed eGFP fluorescence lifetimes comparable to cells expressing eGFP only, indicating that the fluorescence of eGFP was not influenced by an acceptor fluorophore. mCherry-repM^pro^(C145A)-eGFP exhibited a markedly shorter fluorescence lifetime, indicating FRET between eGFP and mCherry embedded in the unprocessed reporter.

### Blocking of proteolytic activity and inhibitor dose-dependent accumulation of precursor M^pro^ can be assessed simultaneously in HEK293T cells

Using flow cytometry, we analyzed cells expressing mCherry-repM^pro^-eGFP to screen a series of inhibitors including both commercially available compounds (boceprevir, GC376, bofutrelvir, ensitrelvir, and nirmatrelvir) and newly designed molecules (compound 1, a nonpeptide inhibitor with an indole trifluoromethylpyridinyl ester moiety; compound 2, a small molecule with glutamine in the P1 position; and compound 3, a peptide derivative prolonged to the P1′ position with an α-ketoamide warhead).

**Figure 6:**
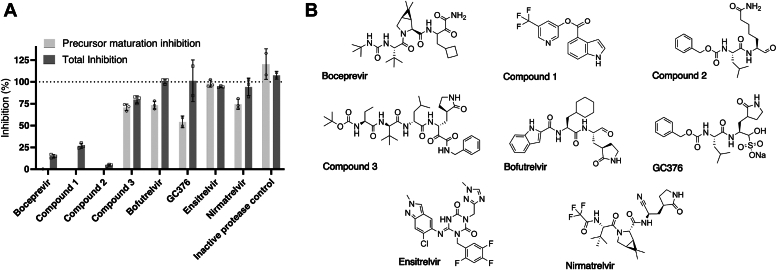
**Testing of a series of inhibitors using the mCherry-repM**^**pro**^**-eGFP reporter**. *A*, HEK293T cells were transiently transfected with plasmid encoding the reporter. Immediately after transfection, the cells were treated with 20 μM of each inhibitor in triplicate. After 24 h, the cells were harvested and analyzed by flow cytometry. The mCherry signal (measured at 610 nm upon excitation at 561 nm) corresponds to the total inhibition of all protease forms (precursor, partially cleaved precursor, and mature form), the FRET signal (measured at 695 nm upon excitation at 488 nm) corresponds to the amount of uncleaved M^pro^ artificial precursor. The signal from the reporter featuring the C145A active site mutation was considered as 100% inhibition. Error bars represent standard errors. *B*, structures of newly designed and known M^pro^ inhibitors used in the screen. M^pro^, main protease.

HEK293T cells were transiently transfected by the reporter plasmid, and compounds of interest were added to a final fixed concentration of 20 μM. After 24 h, the cells were harvested and subjected to flow cytometry (Fig. 6*A*). Inhibition of proteolytic activity was represented by an increase in mCherry signal (excitation at 561 nm, fluorescence at 610 nm). The FRET channel was used to quantify inhibitor dose-dependent accumulation of the uncleaved precursor (excitation at 488 nm, fluorescence at 610 nm). mCherry-repM^pro^(C145A)-eGFP was used as a reference sample with maximal signals in both channels. The lead compounds from the screen (GC376, bofutrelvir, ensitrelvir, nirmatrelvir, compound 1, and compound 3, Fig. 6*B*) were further tested.

We measured fluorescence dose responses of the six selected small molecules within a wide concentration range (Table 1, Fig. S4). The compounds differed in their ability to block overall M^pro^ activity and to retain M^pro^ in its precursor form (Table 1, Fig. S4).

**Table 1:** Comparison of inhibition potencies as determined by the cell-based reporter assay, recombinant protein inhibition, and viral replication assay

	HEK293T cells, reporter mCherry-repM^pro^-eGFP		Anti-SARS-CoV-2 activity in Calu-3 cells EC_50_ [μM]	Toxicity in Calu-3 cells, XTT assay CC_50_ [μM]	Purified recombinant protease in assay with fluorescent peptide substrate	
	Precursor processing inhibition EC_50_ [μM]	Total inhibition EC_50_ [μM]			Precursor preM^pro^ (K_i_ calculated from IC_50_, inhibition phase, maximal signal = activated) [μM]	Mature M^pro^ (Morrison plot, K_i_) [nM]
Nirmatrelvir	7.79 ± 0.83	0.075 ± 0.010	0.77 ± 0.05	>50	16.6 ± 1.6	1.14 ± 0.14
GC376	46 ± 12	0.228 ± 0.038	7.90 ± 0.48	>64	23.0 ± 3.5	3.1 ± 3.1
Compound 1	n.d.	12.1 ± 2.8	>64	>64	n.d.	n.d.
Ensitrelvir	1.474 ± 0.075	0.0070 ± 0.0023	0.104 ± 0.017	>50	16.9 ± 1.3	0.89 ± 0.41
Bofutrelvir	2.52 ± 0.40	0.0901 ± 0.015	5.30 ± 0.36	>64	5.79 ± 0.50	1.24 ± 0.44
Compound 3	4.75 ± 0.24	0.382 ± 0.053	23.0 ± 2.0	>128	8.76 ± 0.57	0.89 ± 0.39
Remdesivir^a^	n.d.	n.d.	0.32 ± 0.03	>64	n.d.	n.d.

Standard errors are reported for each fitted value.

M^pro^, main protease.

a = Remdesivir, the positive control inhibitor in the SARS-CoV-2 viral replication assay, is an inhibitor of RNA-dependent RNA polymerase, not of M^pro^.

In all cases, higher doses of inhibitors were needed to retain M^pro^ in its precursor form than to inhibit the total enzyme activity. The two compounds in clinical use (nirmatrelvir, ensitrelvir) were the best inhibitors of proteolytic activity. However, their abilities to retain the reporter in a precursor form were comparable to those of bofutrelvir and compound 3. These latter two compounds showed lower selectivity between inhibition of overall activity and the ability to cause accumulation of the unprocessed precursor reporter.

### Orthogonal assays confirmed inhibitory activities of the small molecules selected by the cell-based assay

To validate and reassess the data obtained by our cell-based assay, we tested the small molecules using independent, well-established methods, including inhibition of whole virus replication and enzyme kinetics.

First, we tested the compounds in a viral replication assay using Calu-3 cells infected by SARS-CoV-2. This antiviral assay reflects inhibitory potency at the cellular level, including the inhibitor’s cell-membrane permeability and metabolic stability. The antiviral effects of the small molecules correlated best with overall inhibition of proteolytic activity obtained with our cell-based assay (Table 1). For compound 1, we did not observe a decrease in viral titers, suggesting that the micromolar inhibition observed in our reporter assay is not sufficient for antiviral activity. Compound 3 was a submicromolar inhibitor of overall proteolytic activity and showed antiviral inhibition in the micromolar range.

Using a noninfectious fluorescent cell-based assay, we gleaned information about inhibition for compounds that are not sufficiently potent to inhibit SARS-CoV-2 in infected Calu-3 cells. This assay can also illuminate situations in which the antiviral effect originates from anti-M^pro^ activity as well as modulating a host off-target molecule.

The EC_50_s for mCherry-repM^pro^-eGFP processing inhibition were in the micromolar range for most inhibitors tested, which is likely insufficient to contribute to the antiviral effect. The potential influence of precursor processing inhibition was likely overshadowed by overall enzyme activity inhibition at lower concentrations of inhibitors.

To separately assess inhibition of the mature and precursor forms, we employed classical enzyme kinetics experiments with purified mature M^pro^ bearing authentic N and C termini, as well as with a Q-1I preM^pro^ form (Figs. 1*C* and 2*A*). This model precursor showed low, but measurable, activity.

Recombinant proteins were mixed with a fluorescence peptide substrate (Dabcyl-Asn-Arg-Abu-Orn-Leu-Gln-Ser-Gly-Asn-Ser-Arg-Lys-Edans, or SUBcor) and Michaelis–Menten kinetics data were collected (Table 2, Fig. S5).

**Table 2:** Michaelis–Menten kinetics of the recombinant main protease and model precursor preM^pro^

Recombinant enzyme	K_M_ (μM)	k_cat_ (s^-1^)
Mature M^pro^	51.4 ± 5.6	0.51 ± 0.27
Precursor M^pro^	80 ± 13	(3.7 ± 0.37) 10^-4^

The curves are depicted in Fig. S5. Standard errors are reported for each fitted value.

M^pro^, main protease.

Consistent with the results from our fluorescent cell-based assay, enzyme kinetic experiments showed that an order-of-magnitude higher amount of inhibitor is needed to block the precursor form, than mature M^pro^ (Table 1). This highlights how our cell-based assay can provide additional information compared to virological tests by detecting the propensity of tested compounds to act at the precursor processing level even when this inhibition is weaker.

K_i_s for mature M^pro^ obtained from the enzyme kinetics assay were similar for all the compounds. The different behaviors of the compounds in tissue culture experiments reflect their different bioavailability and metabolic stability. From this point of view, ensitrelvir, nirmatrelvir, and bofutrelvir inhibit best. From the point of view of precursor inhibition, bofutrelvir and compound 3 showed lower selectivity between different forms of M^pro^.

### Mechanisms of M^pro^ and preM^pro^ inhibition

**Figure 7:**
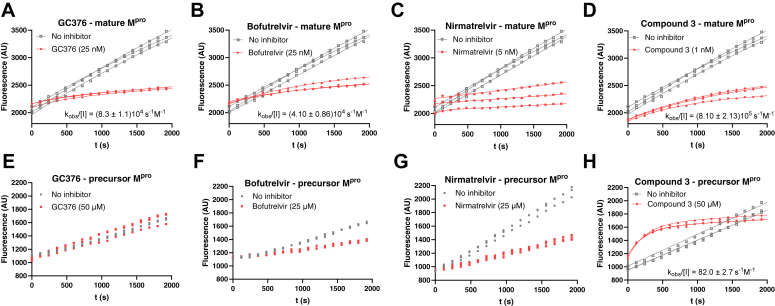
**Examples of the time course of cleavage of peptide substrate in the presence of inhibitors.** Reactions were monitored for 2000 s. The efficacy of some inhibitors was assessed based on the k_obs_/[I] value obtained from fitting the data to the equation for two-step reversible covalent inhibition. The data were obtained for the mature M^pro^ (panels *A*–*D*) and for the recombinant precursor form of M^pro^ (panels *E*–*H*).

Many of the molecules tested are recognized as reversible covalent binders of cysteine proteases. Thus, we followed the time course of cleavage reactions for 2000 s, to determine the effects of potential slow binding (Fig. 7, *A*–*H*). The typical time progress curve is biphasic. The initial linear part corresponds to the initial reaction rate before formation of the covalent bond. After the curve bends over time, the steady-state reaction rate reflects the formation of a covalent, reversible bond between the protease and the inhibitor. We obtained k_obs_ values by fitting the time-course curves to an equation for reversible covalent inhibition. We were unable to calculate k_obs_ for the entire range of inhibitor concentrations, as in some cases, covalent bond formation was likely faster than the onset of measurement. Covalent bonds may also form at a later stage, possibly when the inhibition curve begins to show nonlinearity due to the consumption of the substrate or the instability of the enzyme in the assay.

With this in mind, we selected one suitable inhibitor concentration for evaluation, calculating the k_obs_/[I] parameter wherever possible. As expected, progress curves for substrate cleavage catalyzed by mature M^pro^ in the presence of covalent reversible inhibitors such as GC376 were nonlinear (Fig. 7*A*). In contrast, preM^pro^ with the same inhibitor gave a linear signal response, as evidenced by GC376 inhibition (Fig. 7*E*). The inhibitor could leave the active site before the covalent bond is formed because of its poorer affinity for preM^pro^ than M^pro^. However, more research is needed to establish the mechanism.

**Figure 8:**
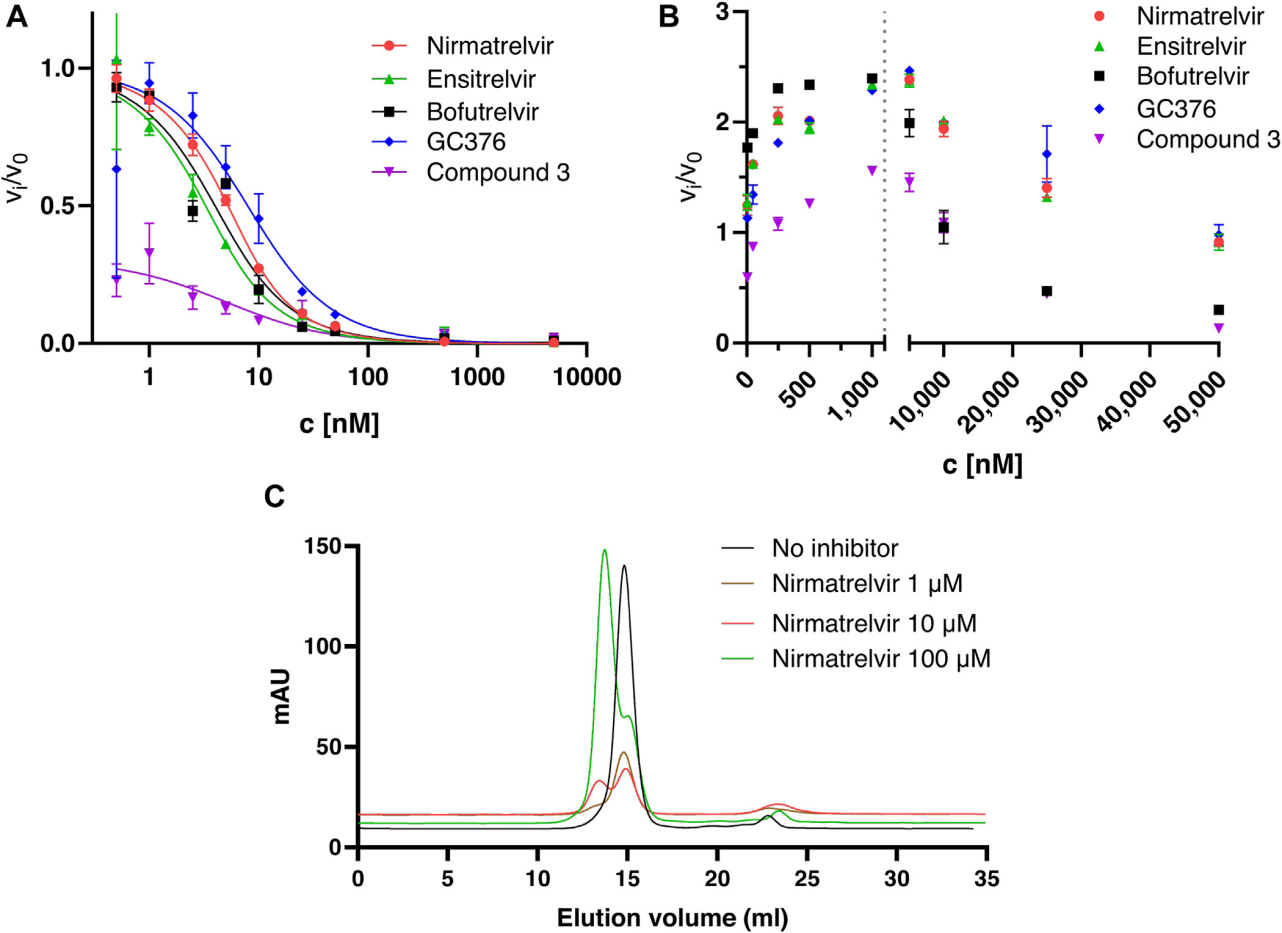
**M**^**pro**^**and preM**^**pro**^**activity in the presence of inhibitors**. *A*, mature M^pro^ inhibition was observed during fluorescent peptide substrate cleavage. The initial reaction rates (inhibited *versus* uninhibited) were plotted against the inhibitor concentrations and evaluated using a Morrison plot (inhibition constants listed in Table 1). Error bars represent SDs calculated from triplicates. *B*, the activity of preM^pro^ was measured in the presence of different inhibitors. Activation (rather than inhibition) was observed at lower inhibitor concentrations. The ratio of initial rates of inhibited reactions *versus* the maximal rate was used to determine IC_50_ values. Error bars represent SDs calculated from triplicates. *C*, dimerization of preM^pro^ induced by nirmatrelvir during analytical chromatography. When a sample of 0.15 mg of preM^pro^ was applied to a Superdex 200-pg column, we observed a peak at around 15 ml. The same amount of preM^pro^ supplemented with nirmatrelvir shifted the peak to 13 ml, depending on the dose of the inhibitor. preM^pro^, precursor form of M^pro^; M^pro^, main protease.

To compare inhibitors of interest, we evaluated substrate cleavage rates after 20 min using the stable parts of the time progress curves. Data obtained for M^pro^ were fitted to the Morrison equation to obtain K_i_ (Fig. 8*A*, Table 1). All the K_i_ values were in the low-nanomolar range, again indicating that differences between inhibitors in cellular assays can be attributed to their pharmacokinetic properties.

K_i_ values for preM^pro^ were obtained as IC_50_/(1+[S]/K_M_) (Table 1). Bofutrelvir and compound 3 showed the best inhibition of preM^pro^. Interestingly, preM^pro^ was predominantly activated by lower concentrations of the compounds (Fig. 8*B*). For evaluation purposes, the curves were split into two parts, indicating the activation and inhibition phases. Only the inhibition parts were evaluated for the comparison of small molecules to obtain IC_50_s.

Activation was observed for every tested small molecule binding to preM^pro^. One hypothesis is that small molecule binding to the preM^pro^ monomer may induce dimerization. M^pro^ dimer is more active than the monomer. As the concentration of the small molecules increases, this activity may reach a maximum. At higher concentrations, the inhibition effect would start to compensate for the activation, leading to an activity decrease.

To investigate the effect of small molecules on dimerization, we performed a series of analytical chromatography experiments using active preM^pro^ and different concentrations of nirmatrelvir. We observed a nirmatrelvir dose-dependent shift of the main peak toward earlier elution time, indicating the formation of the dimer (Fig. 8*C*).

## Discussion

The zymogenic forms of proteolytic enzymes have been studied less extensively than their mature counterparts. Due to the irreversible nature of proteolysis, which often triggers a cascade of downstream events, proteases are typically synthesized as minimally active or inactive zymogens. Specific conditions subsequently trigger the transformation of these zymogens into active enzymes (92). For example, SARS-CoV-2 expresses its main protease in a zymogenic form (Fig. 1*A*), which is activated by processing, dimerization (49, 50), and possibly octamerization (93).

To prepare a model precursor, we mutated the conserved P1 glutamine (Fig. 1*B*) in the N-terminal autoprocessing cleavage site of the M^pro^ precursor (Fig. 1*C*). Most mutations did not entirely block N-terminal processing of the precursor (Fig. 2*A*), in agreement with recent findings (94). The fact that N-terminal processing is blocked by M^pro^-specific inhibitors (Fig. 2, *B* and *C*) indicates that M^pro^ is involved in the cleavage process. Our data using preM^pro^-derived protein substrates confirmed the essential role of glutamine in the P1 position for *trans* cleavage (Fig. 3). Cleavage of those substrates can be blocked by M^pro^-specific inhibitors (Fig. S1). Similar results were obtained with analogous dodecapeptide substrates with P1 substitutions (Fig. S2).

The difference in cleavage behavior *in cis versus in trans* likely reflects differences in steric accessibility of the cleavage site and/or the monomolecular mechanism of *cis* cleavage. Alternatively, precursor substrate-binding pockets may have steric arrangements that differ from the mature form. The strict requirement for glutamine at the natural processing site, a feature conserved among related viruses (1), indicates the importance of an appropriate rate of cleavage and/or a benefit of *trans* cleavage during M^pro^ maturation.

Expression of recombinant variants of preM^pro^ in bacteria yielded insights into their potential cytotoxicity (Fig. 2*A*). When processing of the less active, and thus less toxic, precursor form is decelerated (as in the Q-1A mutant, and particularly in the C145A and Q-1I mutants), overall expression of the recombinant protein is increased compared with WT preM^pro^, where the portion of the cytotoxic mature protease is highest. A similar cytotoxic effect can be seen in HEK298T cells (Figs. 4 and 5*A*), as previously described (26).

Furthermore, we investigated the possibility of targeting preM^pro^ with small molecules. Inhibiting the viral protease in its precursor form offers significant advantages for regulating the viral life cycle. First, intercepting the virus at an earlier stage of its life cycle could prevent the emergence of rescue mutations during antiviral treatment (66, 68, 95). Second, the precursor could *trans* dominantly inhibit the production of mature virions (68, 69, 70, 96). Third, the inhibition of both precursor and mature forms of the protease by distinct molecules and mechanisms presents an opportunity for further exploration, as does the interplay between these cleavage mechanisms. Compounds selectively targeting zymogen and mature protease may yield novel insights into the fundamental understanding of proteases.

Studying potential inhibitors in living cells is beneficial as it directly yields additional information about their cytotoxicity and cell penetration. Our cell-based reporter system (Fig. 4) allowed us to quantify the uncleaved precursor form and measure total protease inhibition. As inhibition is accompanied by a gain-of-fluorescence signal, this assay favors nontoxic inhibitors that do not disrupt the cellular machinery, ensuring production of reporter proteins. This is an advantage over virologic assays in which the antiviral effect is assessed as reduced production of viable viral progeny. However, the interaction of test compounds with host off-targets may also lead to a reduction in the production of viral progeny independently of targeting M^pro^.

We validated this system using a combination of techniques: immunoblotting (Fig. 5*A*), FCCS (Fig. 5*B*), FLIM (Figs. 5*C* and S3), and flow cytometry (Fig. 6*A*). All four methods are applicable for M^pro^ inhibitor evaluation, assessing both their ability to inhibit proteolytic activity and their potential to preserve M^pro^ in its precursor form. As expected, we observed inhibitor dose-dependent accumulation of the fluorescent proteins in transfected cells (Figs. 5*A* and S4).

We screened a series of compounds including both in-house designed and commercially available M^pro^ inhibitors (Fig. 6*A*). The nonpeptide compound 1 was a derivative of indole chloropyridinyl esters (97), whereas compounds 2 and 3 were pseudopeptides. Across multiple assays, the compounds exhibited a greater inhibitory effect on M^pro^ activity than a propensity to trigger accumulation of uncleaved precursor. We confirmed the advantage of cyclization of the P1 glutamine in pseudopeptide inhibitors (compound 2 *versus* compound 3). Intriguingly, certain inhibitors lacking glutamine or its corresponding γ-lactam in the P1 position have been reported. These include boceprevir, which features a cyclobutyl moiety (86); pomotrelvir, containing 2-oxopiperidin (98, 99); and MK-7845, bearing a difluorobutyl substituent (100). Substrates with methionine or histidine in the P1 position also have been reported (101).

Compound 3 and bofutrelvir exhibited smaller differences in potency for M^pro^ and preM^pro^ inhibition than the other small molecules. Nevertheless, their ability to inhibit preM^pro^ at micromolar concentrations was not sufficient to produce an effect in virological experiments. Compound 3, an α-ketoamide, is suitable for Px′ modifications that can enrich the chemical space (102, 103, 104, 105, 106, 107, 108, 109) for further optimization of precursor-specific inhibitors. This supports the involvement of Px′ positions in inhibitor design as a viable strategy, substantiating previous observations (84, 110, 111, 112, 113, 114).

In experiments with purified enzymes, we used fluorescent substrate Dabcyl-Asn-Arg-Abu-Orn-Leu-Gln-Ser-Gly-Asn-Ser-Arg-Lys-Edans (abbreviated as SUBcor). The design was inspired by results obtained from a screen of large-scale substrate library (115). We observed significant differences in the kinetics of mature and precursor M^pro^, mainly in k_cat_ (Table 2). This suggests that the precursor form may exist as a conformational ensemble, with only a small fraction adopting a fold resembling the mature form and exhibiting enzymatic activity.

We examined several small molecules with a reactive warhead interacting with catalytic cysteine of M^pro^ for their ability to act as reversible covalent binders (Fig. 7). Obtaining k_obs_ values for the entire inhibitor concentration range was challenging. For some inhibitors, such as nirmatrelvir, covalent bond formation likely occurred rapidly, before the measurement commenced (Fig. 7*C*). Some other inhibitors probably formed a covalent bond slowly, clouding results due to substrate consumption or enzyme instability. Therefore, we selected a suitable inhibitor concentration to calculate the k_obs_/[I] parameter, a strategy previously adopted for evaluation of human rhinovirus protease inhibitors (116).

Curves for inhibition of precursor were linear throughout the entire course of the measurement. One explanation could be that the onset of equilibrium of covalent bond formation is very fast. Alternatively, and perhaps more likely, the inhibitors are not able to form a covalent bond with preM^pro^ at all. One exception was compound 3 which seemed to activate the precursor in the first phase of the reaction and inhibit it in the latter phase (Fig. 7*H*).

To compare the potencies of inhibitors with different binding modes, we used only the linear parts of progress curves for evaluation. For the mature M^pro^ small molecule pairs, the Morrison equation was used to obtain K_i_ values (Fig. 8*A*, Table 1). As only higher concentrations of the small molecules inhibit precursor, IC_50_ values were used for K_i_ calculations (Table 1). Interestingly, our experiments involving recombinant preM^pro^ at low inhibitor concentrations showed enzyme overactivation (Fig. 8*B*), a phenomenon previously observed for mature M^pro^ and GC376 (55). Binding of GC376 at low concentrations to a single monomeric subunit of M^pro^ may induce formation of a more active dimer, stabilizing the second monomer for more efficient substrate cleavage. Conversely, at higher concentrations, the inhibitor may occupy both active sites within the dimer, blocking the possibility to bind and subsequently cleave the substrate. Our analytical size-exclusion chromatography data suggest that nirmatrelvir treatment induces preM^pro^ dimerization (Fig. 8*C*). This is in an agreement with recent findings that nirmatrelvir, GC373, and ensitrelvir can induce dimerization, and even oligomerization of dimerization defective deletion mutants of M^pro^, accompanied by increased activity at lower inhibitor concentration (117).

However, the activation phenomenon is likely to be more complex (Fig. 7*H*). Activation may occur by an induced-fit mechanism (118), in which compounds binding to the active site facilitate a conformational change into a catalytically suitable form, followed by dissociation of the compound and restoration of the enzyme to the proper fold. This assumption is supported by structural data showing mature M^pro^ structural features in a model precursor bound to GC376 (119). Similar activation by low inhibitor concentrations has also been reported for the hepatitis C virus NS3/4A protease (120). Further research is required to elucidate this phenomenon of activation, which could contribute to development of new therapeutic strategies.

## Conclusions

We demonstrate that some mutations in the first amino acid adjacent to the M^pro^ N terminus (P1 position) do not block N-terminal processing and the release of mature M^pro^ (*in cis* processing). We did not observe such behavior for *trans* cleavage, for which glutamine proved indispensable in the P1 position of both protein and peptide substrates. We exploited a mutation that blocks N-terminal *cis* autoprocessing to prepare a recombinant preM^pro^ model for *in vitro* analysis. We employed a cell-based assay that allowed us to simultaneously evaluate inhibition of proteolytic activity and accumulation of uncleaved preM^pro^. Overall, we found that preM^pro^ was much less sensitive to inhibition, although some compounds showed lower selectivity between mature M^pro^ and preM^pro^ (compound 3, bofutrelvir). We also observed *in vitro* activation of preM^pro^ at low inhibitor concentrations. Compound 3 exhibited the most interesting behavior, switching between initial activation and later inhibition of the precursor form. This unique behavior in which activation is not dependent on concentration, but on time, should be further studied.

## Experimental procedures

### Chemicals, cells, and viruses

GC376 and boceprevir were obtained from Biosynth Carbosynth, bofutrelvir from MedChemExpress, and nirmatrelvir and ensitrelvir from Sigma-Aldrich. Restriction enzymes were obtained from New England Biolabs. Untagged mature M^pro^ was obtained from Sigma-Aldrich. HEK293T and Top10 competent *E*. *coli* cells were obtained from the American Type Culture Collection (ATCC) and Thermo Fisher Scientific, respectively. Competent Rosetta *E*. *coli* (DE3) cells were purchased from Sigma-Aldrich. Vero CCL81 (ECACC 84113001) were obtained from the European Collection of Cell Cultures. Calu-3 cells were obtained from ATCC (HBT-55). All cell lines were routinely tested and confirmed mycoplasma negative at Generi Biotech. Cells were maintained in Dulbecco’s modified Eagle’s medium (DMEM) supplemented with L-glutamine, 10% fetal bovine serum (FBS), 100 U/ml of penicillin, and 100 μg/ml of streptomycin (all from Sigma-Aldrich) at 37 °C with 5% CO_2_. SARS-CoV-2 isolate (hCoV-19/Czech Republic/NRL_6632_2/2020) was obtained from a nasopharyngeal swab by inoculating Vero CCL81 cells in a biosafety level-3 laboratory. The virus was expanded by two additional passages, aliquoted, and then stored at −80  °C.

### Cloning

All clones were verified by sequencing, which was performed by SEQme.

### Cloning of bacterial expression plasmids

The nsp4 fragment was amplified using the upstream primer CCATATCGAAGGTCGTCATATGGTAGTCTTTAATGGTGT and downstream mutagenic primer TAAAACCACTXXXCAAAACAGCTGAGGTG. XXX represents specific codons: CTG for asparagine, AGC for alanine, TTC for glutamic acid, AAA for phenylalanine, TCG for arginine, CTT for lysine, and AAT for isoleucine. The nsp5 (M^pro^) fragment was amplified using the mutagenic upstream primer TGTTTTGXXXAGTGGTTTTAGAAAAATGGC, where XXX corresponds to the aforementioned variants, and the downstream primer GCTTTGTTAGCAGCCGTTATTGGAAAGTAACACCTGAG. The WT variant and the C145A active site mutant were amplified by nonmutagenic primers. The pET16b plasmid was cleaved with *Nde*I and *Bam*HI-HF restriction enzymes. Finally, the amplified fragments were inserted into the cleaved vector using the Gibson assembly method (121).

### Preparation of an expression construct for subsequent purification of preM^pro^

We constructed a pET16b plasmid containing a SUMO-preM^pro^ fusion protein. The SUMO segment was obtained by PCR using the upstream primer CCATATCGAAGGTCGTCATATGGGCAACGATCACATTAACCTGAAAGT and the downstream primer AAGACTACGCTGCCGCTGCTTCCACCGGTCTGTTGCTGGAAC. The preM^pro^ segment was amplified using the upstream primer CGGCAGCGTAGTCTTTAATGGTGTTTCCTTTAGTACTTTTGAAGAAG and the downstream primer GCTTTGTTAGCAGCCGGATCCTCATTATTGGAAAGTAACACCTGAGCATTGT. The resulting fragments were then inserted into pET16b cleaved with *Nde*I and *Bam*HI using the Gibson assembly. The resulting gene for expression coded for a fusion protein with decaHisTag-XaCLS-SUMO-SSGS-99AAnsp4-M^pro^.

### Cloning of the reporter mCherry-repM^pro^-eGFP

The original sequence of SARS-CoV-2 M^pro^, with its N-terminal and C-terminal flanking sequences inserted into pcDNA3.1(+)-C-6His, was ordered from GenScript. The sequence was selected based on viral sequences obtained from patients at the end of January 2020. These sequences are available at GISAID (BetaCoV/Wuhan-Hu-1/2019|EPI_ISL_402125, BetaCoV/Zhejiang/WZ-02/2020|EPI_ISL_404228, BetaCoV/Zhejiang/WZ-01/2020|EPI_ISL_404227, BetaCoV/Nonthaburi/61/2020|EPI_ISL_403962) (https://gisaid.org/). To obtain the active site mutant, the entire plasmid was amplified using the forward primer 5′-GTTCAGCTGGTAGTGTTGGTTTTA and the reverse primer 5′-CATTAAGGAATGAACCCTTAATAGTGA. The WT and C145 A mutant with 10 amino acids adjacent to the N and C termini were amplified with the forward primer 5′-TTTAAGCTTCAAACCTCTATCACCTCA and the reverse primer 5′-TTTGGTACCGTACCCTTGATTGTTCTTT. The PCR products were cleaved with *Hind*III and *Kpn*I restriction enzymes and inserted into the reporter plasmid (67), derived from the pmCherry-C1 vector (Clontech), between mCherry and eGFP.

### Bacterial expression and purification

For small-scale expression, pET16b encoding the protein of interest was transformed into chemically competent Rosetta *E*. *coli* (DE3) cells (Sigma-Aldrich). The transformed cells were placed on LB agar plates containing 40 μl/ml kanamycin and incubated overnight at 37 °C. A single colony was inoculated into a 50 ml Falcon tube containing LB medium supplemented with 40 μg/ml ampicillin and incubated at 37  °C until the culture reached an *OD*_600_ of 0.6. The bacteria were subsequently induced with 1 mM IPTG and incubated overnight at 20  °C in the presence or absence of an M^pro^ inhibitor. On the third day, 200 μl of the bacterial suspension were mixed with 40 μl of SDS sample buffer, heated to 95  °C for 3 min, and processed by SDS–PAGE electrophoresis.

For large-scale expression, bacterial culture was grown in a 2.5 L Erlenmeyer flask. Bacteria were harvested by centrifugation at 6000×*g* for 15 min, resuspended in lysis buffer (50 mM Tris–HCl, pH 8.0, 300 mM NaCl) and lysed using a CF1 cell disruptor (Constant Systems) at a processing pressure of 15 to 20 kpsi. After centrifugation (20,000×*g* for 30 min), the supernatant was loaded onto Ni-NTA resin (Roche) for protein purification. Following a 2-h incubation to enable protein binding, the resin was washed with 10 column volumes of wash buffer (50 mM Tris–HCl, pH 8.0, 300 mM NaCl, 20 mM imidazole) and eluted for 1 h (50 mM Tris–HCl, pH 8.0, 300 mM NaCl, 250 mM imidazole). This procedure was repeated three times. Combined elution fractions were dialyzed against 50 mM Tris–HCl, pH 7.5, 150 mM NaCl, 14 mM mercaptoethanol. SUMO-tag was removed during dialysis by ubiquitin-like-specific protease 1. Finally, the elution fraction was further purified using a HiLoad 16/600 Superdex 200 pg (Cytiva) chromatographic column.

### Analytical chromatography

Dimerization analysis was performed using analytical chromatography. Protein samples, either with or without inhibitors, were loaded onto a calibrated Superdex 200 10/300 GL column using a 500-μl loop.

### SDS–PAGE analysis

Samples were boiled at 95 °C for 5 min. A 10 μl aliquot of each sample was loaded onto a 14 or 16% polyacrylamide gel, together with 2 μl protein marker (all-blue, Bio-Rad). Electrophoresis was performed for 40 min at 200 V. After electrophoresis, gels were either transferred for Western blotting or stained with Coomassie Brilliant Blue G-250 (Serva). Proteins were visualized using a LI-COR Odyssey CLx imager.

### Immunoblot analysis

After electrophoresis, separated proteins were transferred to either nitrocellulose membranes for antibody-based visualization or polyvinylidene difluoride membranes rinsed with methanol for N-terminal Edman analysis using wet electroblotting at 100 V for 1 h. Nitrocellulose membranes were subsequently blocked with casein-based blocking buffer (Thermo Fisher Scientific) with the addition of primary antibodies (RFP antibody 6G6, Chromotek, final dilution 1,500x; GFP antibody ab6556, Abcam, final dilution 1,700x; β-actin antibody A5441, Sigma-Aldrich, final dilution 500,00x; SARS-CoV-2 M^pro^ antibody #51661, Cell Signaling, final dilution 2,000x) or anti-HisTag iBodies (final dilution 2,500x) (122) and incubated overnight at 4  °C. The next day, the membranes were washed three times for 5 min each using tris-buffered saline (TBS)-T (0.1% Tween-20), followed by a single 5 min wash using TBS. Incubation with a secondary antibody (IRDye 800CW goat anti-rabbit IgG and IRDye 680RD goat anti-mouse IgG, Li-Cor Biotechnology, final dilution 100,00x) was then performed for 1 h. Finally, the membranes were washed again (three times for 5 min each with TBS-T and once for 5 min with TBS) and visualized using a LI-COR Odyssey CLx imager.

### N-terminal protein sequencing

Following SDS–PAGE electrophoresis, purified protein samples were transferred onto a polyvinylidene difluoride membrane for blotting. The membrane was stained with 0.5% Ponceau red in 1% acetic acid to visualize protein bands. Bands of interest were excised and analyzed by Edman N-terminal sequencing using the Procise 494 cLC Protein Sequencing System (Applied Biosystems).

### Transfections

HEK293T cells were seeded into 24-well plates and incubated overnight in a growth medium consisting of high-glucose DMEM without L-glutamine and supplemented with sodium pyruvate (Biosera), 10% FBS (Gibco), and 4 mM L-glutamine (Sigma-Aldrich). The cells reached a confluency of 30 to 40% before transfection. For transfection, 0.5 μg (5 μl) of the mammalian expression plasmid was mixed with 1.5 μl of linear polyethylenimine (M_W_ 25,000, 1 mg/ml, Polysciences) diluted in 20 μl of Gibco Opti-MEM medium (Thermo Fisher Scientific) per well. After a 15-min incubation at room temperature, the transfection mixture was added to the cell culture. Subsequently, inhibitors of interest were added at an appropriate inhibitor concentration in 1 μl dimethyl sulfoxide to achieve final concentrations of 100, 25, 6.3, 1.6, 0.4, 0.1, and 0 μM in the culture wells. Cells were harvested after 24 h.

### Fluorescence confocal microscopy (FCCS and FLIM)

An laser scanning confocal microscope (LSM) 780 (Zeiss) was equipped with a 40 × /1.2 water objective and an LSM upgrade kit for time-correlated single photon counting acquisition (Picoquant). Behind the pinhole, emission light split onto two tau-SPAD detectors (Picoquant) with 525/45 (for eGFP) and 600/52 (for mCherry) band pass filters (Semrock). An Intune laser (Zeiss) was used to excite eGFP with a 490 nm wavelength at 40 MHz repetition frequency, and a solid-state continuous wave laser with a 561 nm wavelength was used for mCherry excitation.

FCCS correlation curves were calculated using a previously described algorithm (123) and developed in Matlab (Mathworks). Splitting of the red channel signal according to the fluorescence decay pattern was necessary to avoid detector crosstalk (124).

Fluorescence decays in each pixel were transformed to the phasor diagram. The FLIM data was then taken from the largest population of pixels with identical decay function.

### Flow cytometry

Expression of different variants of fluorescent proteins was analyzed 24 h after transfection using a BD LSRF Fortessa flow cytometer (BD Biosciences). The data were processed with BD FACSDiva 8.0.1 software (BD Biosciences; https://www.bdbiosciences.com/en-us/products/software/instrument-software/bd-facsdiva-software). The signal from positive single living cells was measured in the following channels: EGFP (488 nm/530 nm), mCherry (561 nm/610 nm), and mCherry-preM^pro^-eGFP (488 nm/695 nm). Due to a partial overlap of signals in the 488 nm/695 nm channel, the signal in the FRET channel was corrected as previously described (67). The FRET-corrected signal was then calculated using formula Equation 1, with the x-factor and y-factor calculated using formula Equation 2 and formula Equation 3, respectively.

(1) $$F_{\text{cor}}^{{\text{mCherry}-\text{pre}M^{\text{pro}}-\text{eGFP}}^{\frac{488}{695}}}=F^{{\text{mCherry}-\text{pre}M^{\text{pro}}-\text{eGFP}}^{\frac{488}{695}}}-{\text{xF}}^{{\text{mCherry}-\text{pre}M^{\text{pro}}-\text{eGFP}}^{\frac{488}{530}}}-yF^{{\text{eGFP}}^{\text{mCherry}-\text{pre}M^{\text{pro}}-{\text{eGFP}}^{\frac{561}{610}}}}$$

where

(2) $$x=\frac{F^{{\text{eGFP}}^{488/695}}}{F^{{\text{eGFP}}^{488/530}}}$$

(obtained from cells transfected with pEGPP-N1),

(3) $$y=\frac{F^{{\text{mCherry}}^{488/695}}}{F^{{\text{mCherry}}^{561/610}}}$$

(obtained from cells transfected with pmCherry-C1)

EC_50_ values, defined as the compound concentration resulting in a 50% reduction in signal, were determined using nonlinear regression analysis. The analysis was carried out on plots of percentage signal *versus* log_10_-transformed drug concentration, generated in GraphPad Prism version 10 for Windows (GraphPad Software; https://www.graphpad.com/).

### Antiviral activity

Anti-SARS-CoV-2 activity was measured by determining the extent to which the test compounds inhibited virus replication in Calu-3 cells (HBT-55, ATCC). Briefly, two-fold serial dilutions of compounds were added in triplicate to a 384-well plate with 15,000 Calu-3 cells plated in a DMEM medium with 2% FBS, 100 U of penicillin/ml, and 100 μg of streptomycin/ml (all Merck). After a 1-h incubation, SARS-CoV-2 was added at a multiplicity of infection of 0.02 IU/cell. After 3 days of incubation at 37  °C in 5% CO_2_, cell viability was determined by the addition of an XTT solution (Sigma-Aldrich) followed by incubation for 4 h. The absorbance of the newly formed orange formazan solution was measured using an EnVision plate reader (PerkinElmer). Compound concentrations resulting in a 50% reduction in absorbance (EC_50_) were calculated as described above.

### Cytotoxicity assay

The cytotoxicity of the compounds was evaluated by incubating the same serial dilutions of each compound with Calu-3 cells. Following incubation at 37  °C in a 5% CO_2_ incubator for 3 days, cell viability was determined as above. The compound concentrations resulting in a 50% reduction of absorbance (CC_50_) were calculated as described above.

### Recombinant protein activity assay

Reactions were conducted on a 384-well plate with chimney profile (Greiner) in a final volume of 25 μl of reaction buffer (20 mM Tris–HCl, pH 7.5, 200 mM NaCl, 1 mM EDTA, 1 mM DTT, 10% glycerol, 1% dimethyl sulfoxide). Mature M^pro^ (6 nM by absorbance at 280 nm) or preM^pro^ (9.5 μM) were added to buffer containing an appropriate concentration of inhibitors and/or substrate (Dabcyl-Asn-Arg-Abu-Orn-Leu-Gln-Ser-Gly-Asn-Ser-Arg-Lys-Edans, where Abu stands for α-aminobutyric acid and Orn stands for ornithine). To obtain a sufficiently soluble compound, we designed this substrate using data from a substrate library screen (115). For k_cat_/K_M_ measurement, substrate concentrations ranged from 0 to 80 μM. Higher concentrations were avoided to prevent the inner filter effect. Cleavage of the substrate at 30 °C was monitored using Tecan Spark for 30 min with an excitation wavelength of 340 nm and a detection wavelength of 490 nm. Reactions with inhibitors were measured for 180 min and contained 50 μM substrate.

As many of the inhibitors tested are reversible covalent binders of the active site, we evaluated their potencies by fitting nonlinear curves from the first 1 h of cleavage to slow binding Equation 4. The reaction can be approximated in two steps.

(4) $$E+I\overset{k_{1}}{\underset{k_{-1}}{↔}}EI\overset{k_{2}}{\underset{k_{-2}}{↔}}EI^{*}$$

(5) $$K_{I}=\frac{k_{-1}}{k_{1}}$$

(6) $$P\left(t\right)=P_{0}+V_{S}t-\frac{\left(V_{s}-V_{0}\right)\left(1-e^{-k_{obs}t}\right)}{k_{obs}}$$

(7) $$k_{obs}=\frac{k_{2}\left[I\right]}{K_{I}+\left[I\right]}$$

where *P(t)* is the fluorescence at time *t*, *P*_*0*_ is the background signal at *t* = 0, *V*_*0*_ stands for the initial velocity, *V*_*s*_ is final steady-state velocity, and *k*_*obs*_ is the apparent first-order rate constant representing the equilibrium between EI and EI∗ (125). Covalent inhibitors were evaluated according to the k_obs_/I parameter (116).

In the case of ensitrelvir, a noncovalent binder, we used a standard Morrison plot. After 2000 s, a linear regression can be used to calculate velocity, and relative velocity can be plotted against the inhibitor concentration.

### Chemical syntheses

All syntheses are described in the Supplementary information.

## Data availability

All data supporting this study are reported within this article. Raw data are available from the corresponding author upon reasonable request. LC-MS data are available on https://doi.org/10.6084/m9.figshare.26574340.v1. Enzyme kinetics data are deposited in the STRENDA database under DOI numbers 10.22011/strenda_db.IZ7U7A, 10.22011/strenda_db.EGLZ6S, 10.22011/strenda_db.ZGLADL, 10.22011/strenda_db.YYUIWE.

Further information and requests for resources and reagents generated in this study are available from the Lead Contact with a completed Materials Transfer Agreement.

## Supporting information

This article contains supporting information.

## Conflict of interest

The authors declare that they have no conflicts of interest with the contents of this article.
