# Supplementary material for: The zymogenic form of SARS-CoV-2 main protease: A discrete target for drug discovery
Source: J Biol Chem. 2024 Dec 14;301(1):108079. doi: 10.1016/j.jbc.2024.108079 (PMC11773056; doi:10.1016/j.jbc.2024.108079)
Supplement: Supporting Information [file mmc1.docx]

**The zymogenic form of SARS-CoV-2 main protease: A discrete target for drug discovery**

Pavel Novotný^a,b^, Jana Humpolíčková^a^, Veronika Nováková^a,c^, Stancho Stanchev^a^, Kvido Stříšovský^a^, Michala Zgarbová^a,c^, Jan Weber^a^, Robin Kryštůfek^a,b,^, Jana Starková^a^, Martin Hradilek^a^, Adéla Moravcová^a,d^, Jana Günterová^a^, Kathrin Bach^a,c^, Pavel Majer^a^, Jan Konvalinka^a,e^, Taťána Majerová^a^*

^a^Institute of Organic Chemistry and Biochemistry of the Czech Academy of Sciences, Flemingovo nám. 2, 166 10 Prague 6, Czech Republic

^b^Department of Physical and Macromolecular Chemistry, Faculty of Science, Charles University in Prague, 128 43 Prague, Czech Republic.

^c^Department of Genetics and Microbiology, Faculty of Science, Charles University in Prague, 12843, Prague, Czech Republic.

^d^Department of Biochemistry and Microbiology, University of Chemistry and Technology Prague, 166 28 Praha 6, Czech Republic.

^e^Department of Biochemistry, Faculty of Science, Charles University in Prague, 12843, Prague, Czech Republic.

*corresponding author

**Content of Supplementary information:**

Supplementary abbreviations

Supplementary figures

Supplementary methods

Supplementary references

**Supplementary abbreviations**

DCM dichloromethane

DIC diisopropylcarbodiimide

DIEA diisopropylethylamine

DMF dimethylformamide

EtOAc ethylacetate

Fmoc-AA Fmoc-protected amino acid

HOBt hydroxybenzotriazole

iPrOH *iso-*propyl alcohol

LiHMDS **li**thium **h**exa**m**ethyl**d**i**s**ilazide

MeOH methanol

tBuOH *tert-*butyl alcohol

TFA trifluoroacetic acid

TFE trifluoroethanol

**Supplementary figures**

**Supplementary figure S1: Inhibition of cleavage of substrate Q and substrate N by M^pro^ and preM^pro^ in the presence of nirmatrelvir (NMV) and ensitrelvir (ENS).** Cleavage was monitored for 5 days. Samples were analyzed by SDS-PAGE, and gels were stained by Coomassie Brilliant Blue G-250 (Serva). **(A)** Scheme of the active site mutant precursor M^pro^(C145A), named substrate Q. Substrate Q was monitored for cleavage in the presence of 5 or 500 μM NMV (top gel) or 5 or 500 μM ENS (bottom gel) by mature M^pro^ **(B)** or preM^pro^ **(C)**. The last three lanes on the gel in panel C show a control with preM^pro^ only. **(D)** Scheme of a precursor M^pro^(C145A Q-1N), further labeled as substrate N. The construct possesses the active site C145A mutation, canonical glutamine in P1 position of the N-terminal cleavage site is replaced by asparagine. Cleavage of substrate N by mature M^pro^ **(E)** or preM^pro^ **(F)** in the presence of 5 or 500 μM NMV (top gel) or 5 or 500 μM ENS (bottom gel). Cleavage was not observed in any case after addition of SARS-CoV-2 M^pro^ specific inhibitors.


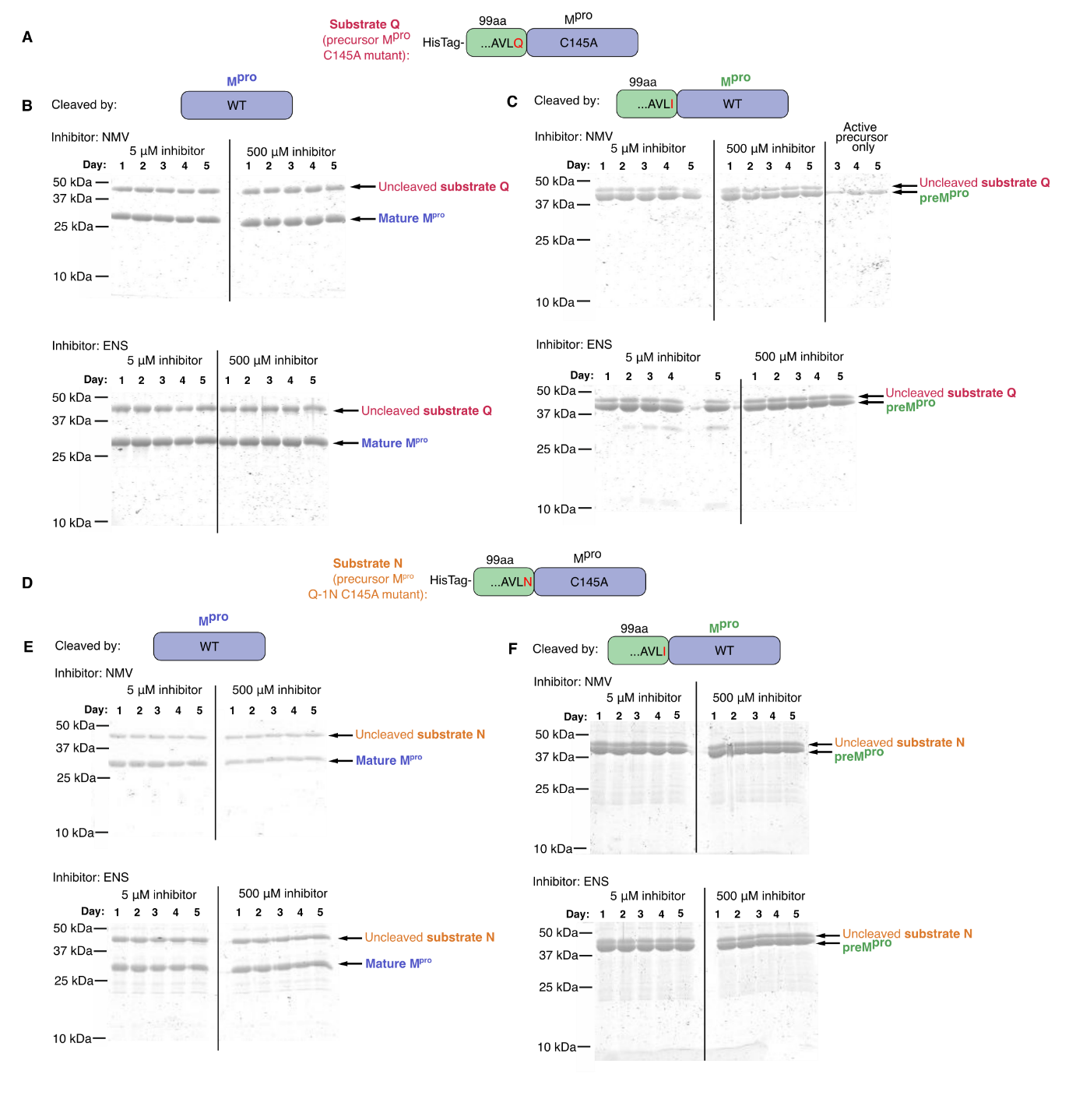


**Supplementary figure S2: Cleavage of peptides with amino acid replacements in the P1 position.** Experimental triplicates were used to calculate mean and standard deviation. The dodecapeptides span six amino acids from each side of the scissile bond between nsp4 and M^pro^. Glutamine in the P1 position, corresponding to the Q-1 position of preM^pro^, was left intact **(A)** or substituted with asparagine **(B)** or alanine **(C)**. We mixed 200 μM of each peptide substrate with 200 nM of mature M^pro^ or 1 μM of preM^pro^ and performed LC-MS analysis after 5 days of cleavage. Peptide substrates and peptides corresponding to proposed products of cleavage were used as reference samples. Only the wild-type peptide substrate was cleaved by mature or precursor M^pro^. AUC on the y-axis indicates the area under the curve of the peak from the extracted ion chromatogram (EIC). In contrast to processing of the recombinant precursor proteases bearing P1 mutations, we did not observe atypical cleavage of the peptide substrates with analogous mutations.


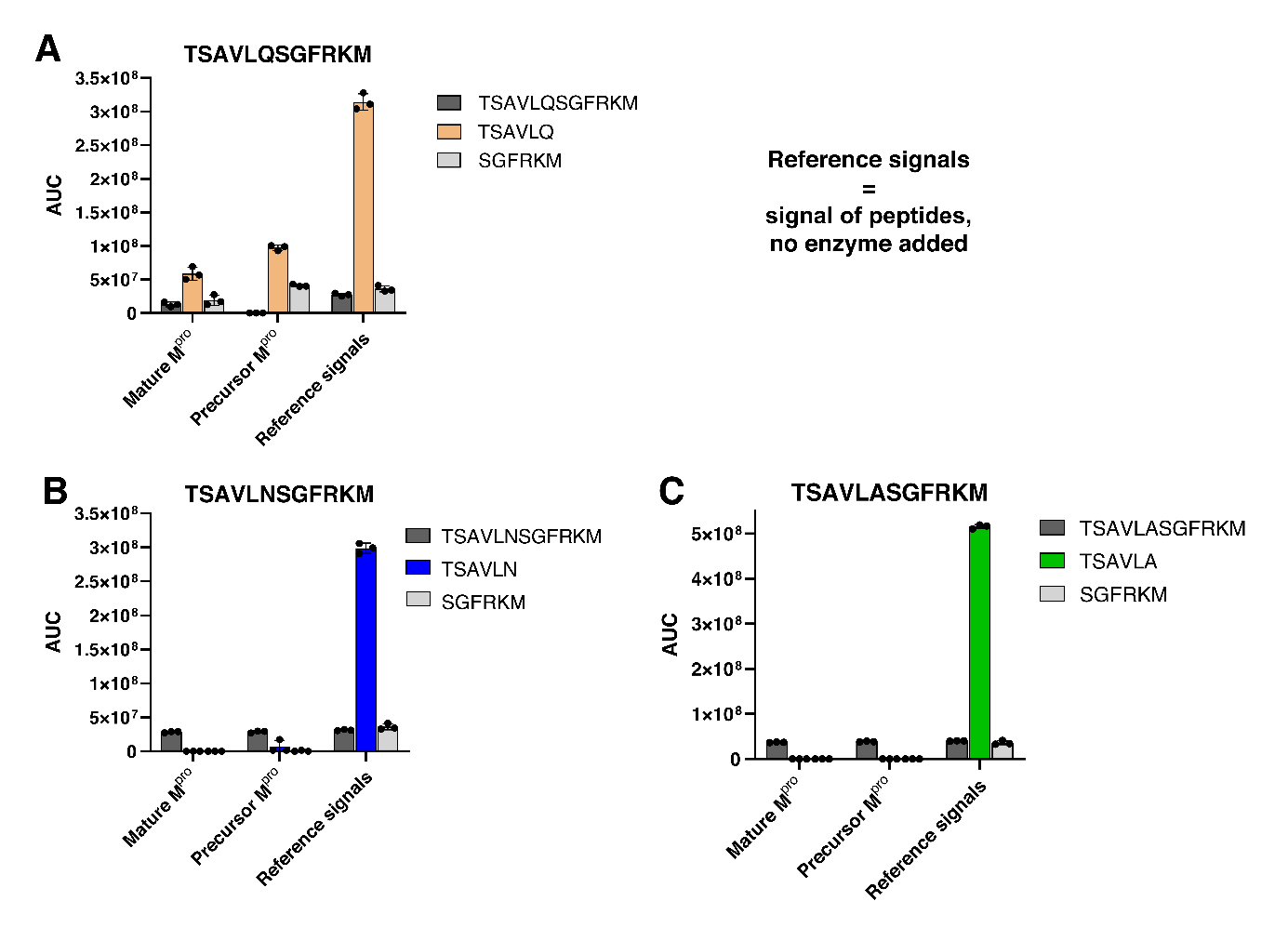


**Supplementary figure S3:** HEK293T cells (from *left* to *right*) transfected by the reporter mCherry-repM^pro^-eGFP **(WT)**, its variant with the C145A active site mutation (mCherry-repM^pro^(C145A)-eGFP, **C145A**), and with plasmid pEGFP-N1 encoding only eGFP **(donor)**. The scale bar indicates 5 µm. Color scale represents the fluorescence lifetime of eGFP excited at 488 nm. The lifetime of eGFP embedded in uncleaved C145A is shorter than the fluorescence lifetime measured in cells with processed WT or cells expressing eGFP only. The reduction in fluorescence lifetime is a result of Förster resonance energy transfer (FRET) to the mCherry moiety, which is in sufficient proximity to the eGFP emitter in uncleaved reporter.


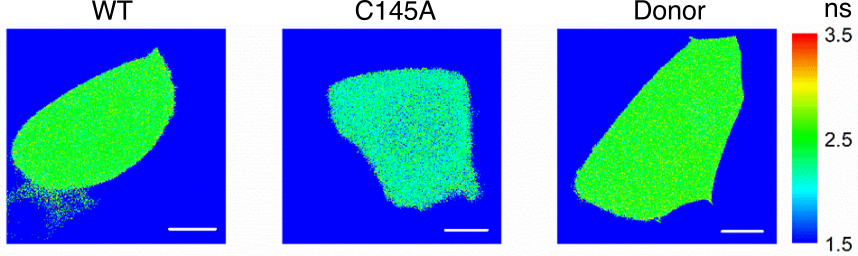


**Supplementary figure S4:** Inhibitor dose-response curves obtained using the reporter mCherry-repM^pro^-eGFP. HEK293T cells were transiently transfected with the plasmid bearing the reporter and treated with appropriate inhibitor concentration. After 24 h, the cells were harvested. The inhibitor dose-dependent increase in intracellular fluorescence was measured by flow cytometry. The signal of mCherry (excitation 561 nm/emission 610 nm) is proportional to the total inhibition of all protease forms, whereas the FRET signal corresponds to the uncleaved mCherry-repM^pro^-eGFP (488 nm/695 nm). Signals of mCherry-repM^pro^-eGFP with the C145A active site mutation served as a reference for 100% inhibition. Cytotoxic concentrations of inhibitors were not taken into the regression. Error bars indicate standard deviations. EC50 values are reported in Table 1.


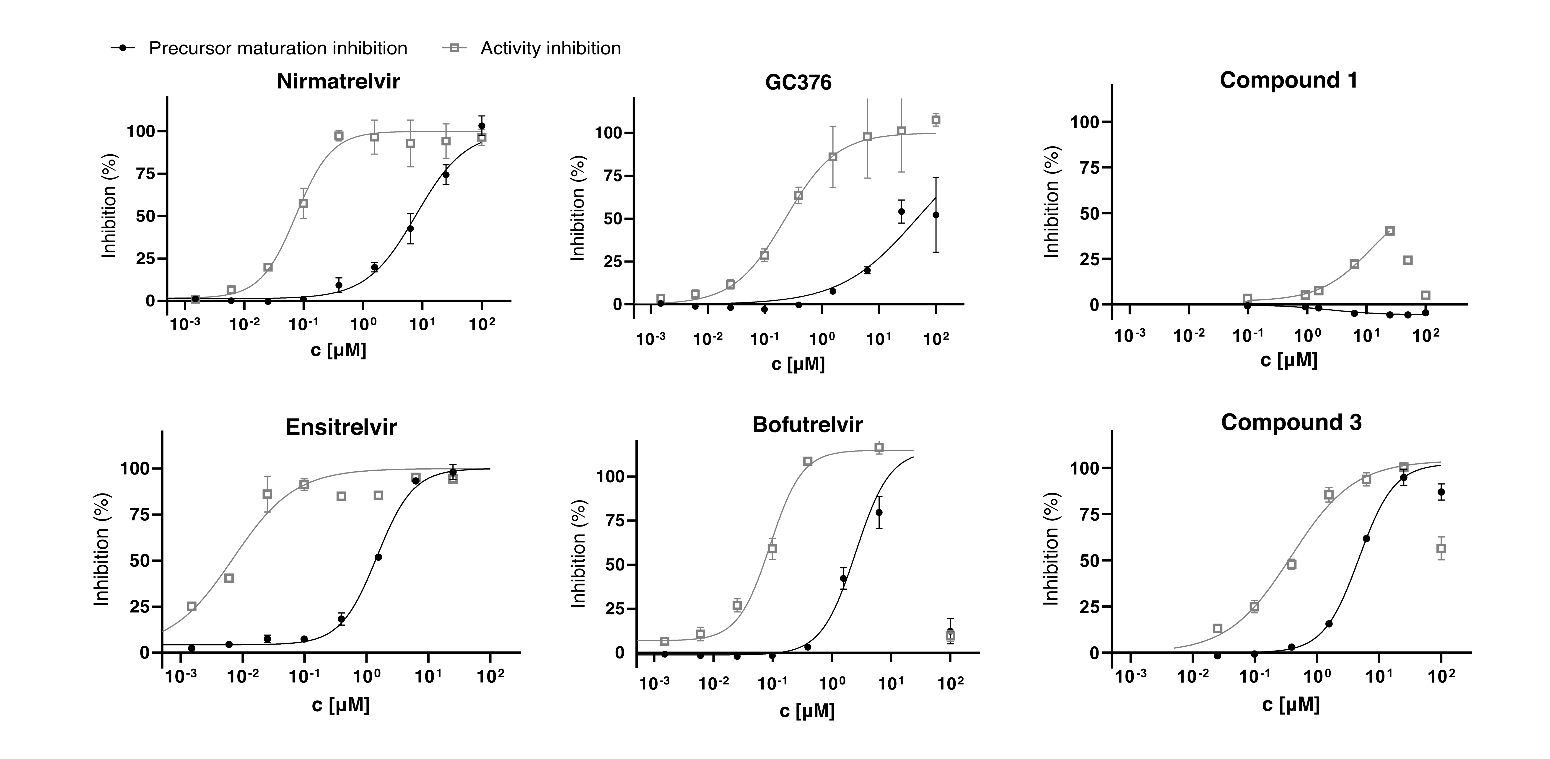


**Supplementary figure S5:** Michaelis-Menten kinetics of mature M^pro^ **(A)** and preM^pro^ **(B)**. Constants are provided in Table 2. Error bars indicate standard deviations. Fitted constants are reported with their standard errors.


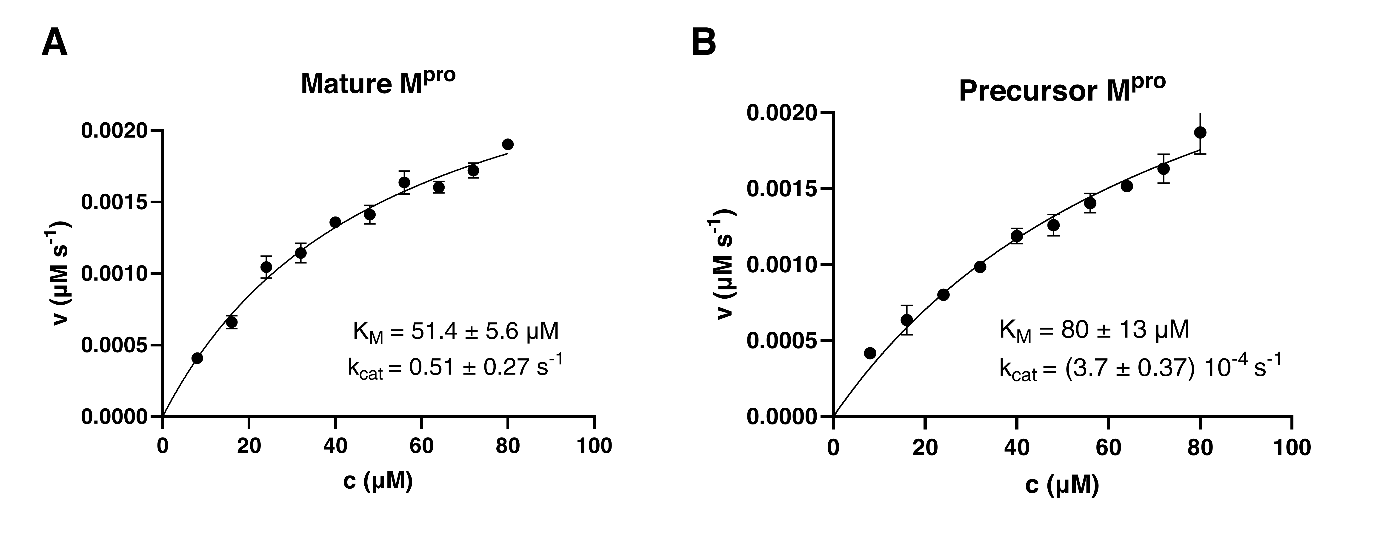


***Supplementary methods – Chemical syntheses***

***General chemical methods***

HPLC analysis and purification were performed using a Jasco PU-1580 Series instrument (Jasco, Japan) equipped with a preparative reversed-phase C_18_ column (250 × 20 mm, 10-μm particle size; Watrex International, Inc, San Francisco, California, USA). The mobile phases consisted of water with 0.1% trifluoroacetic acid (solvent A) and acetonitrile (solvent B). Separation was achieved at a flow rate of 10 mL/min, with elution monitored by absorption at 210 nm using a UV–VIS 1575 detector. Fractions containing the major peak, corresponding to the desired peptide, were combined, frozen, and lyophilized to yield the pure peptide. Analytical runs were performed on the same Jasco module with a Watrex C_18_ column (250 × 4.6 mm, 5-μm particle size) at a flow rate of 1 mL/min. A linear gradient, ranging from solvent A to solvent B over a period of 30 min at room temperature, was used for elution.

For LC–MS analysis, an Agilent Technologies Liquid Chromatograph, coupled with a TOF 6230 ESI–MS detector, was used with a linear gradient with the following parameters: 2% B to 100% B over a period of 10 min on a 1.7-µm particle size C_18_, 100 × 2.1 mm reversed-phase HPLC column (Waters) at a flow rate of 0.3 mL/min, where A = water + 0.1% FA and B = ACN + 0.1% FA. NMR spectra were acquired on a Bruker AV-400 MHz instrument at room temperature.

***Synthesis of compound 1 (5-(trifluoromethyl)pyridin-3-yl 1H-indole-4-carboxylate)***

Compound 1 was synthesized by the esterification (1) of 3-hydroxy-5-(trifluoromethyl)pyridine and indole-4-carboxylic acid, mediated by DIC and N,N-dimethylaminopyridine (DMAP) at room temperature in dry CH_2_Cl_2_. The compound was purified by reversed-phase HPLC using H_2_O with 0.05% TFA and methanol as solvents. HRMS (ESI) (m/z): calculated for C_15_H_8_O_2_N_2_F_3_: 305.05434, found: 305.05380 [M-H]

***Synthesis of compound 2 (benzyl ((S)-1-(((S)-5-amino-1,5-dioxopentan-2-yl)amino)-4-methyl-1-oxopentan-2-yl)carbamate)***

Synthesis of compound 2 was conducted on Thr-Gly resin (2) from L-glutaminal obtained by oxidation of L-glutaminol using polystyrene-immobilized 2-iodoxybenzoic acid (3). The product was then purified by reversed-phase HPLC using a water/acetonitrile gradient with 0.05% TFA as an additive. Finally, the purified compound was repeatedly lyophilized from acetic acid solution to remove TFA, yielding (52%) of compound 2. HRMS (ESI) (m/z): calculated for C_19_H_27_O_5_N_3_Na: 400.18429, found: 400.18399 [M+Na].

***Synthesis of compound 3***

***Synthesis of compound 3.1*** ***(dimethyl (tert-butoxycarbonyl)-L-glutamate) – Scheme 1***

**Scheme 1. Synthesis of compound 3.1 (4).**

Dimethyl glutamate hydrochloride (1.0 g, 4.73 mmol, 1 eq.) was suspended in 15 mL dioxane, then DIEA (1.21 mL, 7.1 mmol, 1.5 eq.) was added until dimethylglutamate was dissolved completely. BOC – anhydride (1.03 g, 4.7 mmol, 0.99 eq.) was added as a solution in 12 mL of dioxane, after which the reaction mixture was stirred at ambient temperature overnight. Dioxane was subsequently removed by evaporation. The residue was dissolved in ethyl acetate (EtOAc) and washed with sat. NaHCO_3_ (2×), 10% KHSO_4_ (2×), and brine (1×). The EtOAc layer was dried over MgSO_4_, with the latter filtered off. EtOAc was removed by evaporation, yielding the crude product. Yield: 1.27 g (98%). ^1^H NMR (401 MHz, DMSO) δ (ppm) = 7.28 (d, *J* = 7.9 Hz, 1H, CONH), 4.07–3.94 (m, 1H, CH), 3.62 (s, 3H, COOCH_3_), 3.58 (s, 3H, COOCH_3_), 2.43–2.32 (m, 2H, CH_2_), 1.93 (dd, *J* = 13.1, 7.8 Hz, 1H, CH_2_), 1.85–1.72 (m, 1H, CH_2_), 1.37 (s, 9H, BOC).

***Synthesis of compound 3.2 ((2S)-2-(tert-butoxycarbonylamino)-3-[(3'S)-2'-oxo-3'-pyrrolidinyl] propanoic acid methyl ester) – Scheme 2***

**Scheme 2. Synthesis of compound 3.2 (5, 6).**

BOC-L-dimethyl glutamate (3.1, 1.25 g, 4.54 mmol, 1.00 eq.) was deposited in a flame-dried round-bottomed flask under nitrogen, to which 13 mL of freshly distilled THF was added. The oily starting material was dissolved at rt, then LiHMDS (1.0 M in THF, 9.9 mL, 7.82 mmol, 2.16 eq.) was added over a period of 2 min at –78 °C. The reaction mixture went from clear and nearly colorless to light yellow and was allowed to stir at –78 °C for 1 h (5, 6). Next, BrCH_2_CN (0.340 mL, 4.86 mmol, 1.07 eq.) was slowly added over a period of 1 h (addition rate of approximately 0.02 mL/5 min), while maintaining the reaction temperature at –78 °C. The reaction mixture was then stirred at –78 °C over a period of 2 h. After 2 h, some starting material remained, but the reaction was quenched by the addition of 7.8 mL of HCl at –78 °C. The reaction mixture was then removed from the cooling bath to allow the ice in the flask to melt and stirred for 50 min. The mixture was then extracted with EtOAc (3×). The combined EtOAc layers were washed with H_2_O (2×) and brine (1×). Drying over MgSO_4_ and concentration by rotavap furnished a dark brown oil. This oil was then dissolved in 7.0 mL of DCM, to which activated charcoal (0.44 g) and silica (1.75 g) were added. The slurry was spun on a rotavap without heat or vacuum for 1 h. Afterwards, the charcoal and silica were filtered through a Celite pad, and the filtride washed with additional volumes of DCM. Concentration of the filtrate furnished a yellow oil. This oil was then transferred to a flame-dried round-bottomed flask under nitrogen, to which CoCl_2_•6H_2_O (0.35 g, 1.46 mmol) was added. The material was then dissolved in 20.0 mL of freshly distilled MeOH. The solution was then cooled to 0 °C and NaBH_4_ (0.56 g, 14.8 mmol) was added in multiple portions over a period of 30 min. Upon the addition of NaBH_4_, the reaction mixture immediately turned black and started bubbling. Once the addition was finished and bubbling slowed, the reaction mixture was capped under a blanket of pressure-equalizing nitrogen, removed from the ice bath, and allowed to warm to room temperature and stirred for 24 h. After 24 h, the reaction mixture was concentrated on a rotavap to a minimal volume. To the obtained residue, 1 M of citric acid at 0 °C was then added. The mixture was further diluted with EtOAc and extracted with EtOAc (3×). The combined EtOAc layers were then washed with sat. NaHCO_3_ (2×) and brine (1×). Drying over MgSO_4_ and the removal of solvent furnished a very pale-yellow oil as the crude product. This material was then purified via flash column chromatography using an eluent system of EtOAc (ninhydrin staining). Yield: 140 mg (11%). ^1^H NMR (401 MHz, DMSO) δ (ppm) = 7.63 (s, 1H, CONH), 7.41 (d, *J* = 8.1 Hz, 1H, CONH), 4.07–3.96 (m, 1H, CH), 3.62 (s, 3H, COOCH_3_), 3.18–3.08 (m, 2H, CH_2_), 2.33–2.21 (m, 1H, CH_2_), 2.19–2.07 (m, 1H, CH_2_), 2.04–1.92 (m, 1H, CH), 1.69–1.48 (m, 2H, CH_2_), 1.38 (s, 9H, BOC). ^13^C NMR (101 MHz, DMSO) δ (ppm) = 178.0, 172.9, 155.6, 78.2, 51.8, 37.7, 32.3, 28.2, 27.2.

***Synthesis of compound 3.3 (tert-butyl ((S)-1-hydroxy-3-((S)-2-oxopyrrolidin-3-yl)propan-2-yl) carbamate) – Scheme 3***

**Scheme 3. Synthesis of compound 3.3 (5, 7).**

Compound 3.2 (0.127 g, 0.44 mmol, 1.0 eq.) was deposited in a flame-dried round bottom flask under nitrogen and dissolved in 2.7 mL of dry THF. LiBH_4_ (4.0 M in THF, 0.33 mL, 1.32 mmol, 3.0 eq.) was then added dropwise (5) to the reaction mixture. Initially, the addition caused the reaction mixture to turn bright yellow. Continued addition eventually resulted in a fading of the yellow color. Gas evolution occurred. After the gas evolution ceased, 1.33 mL of dry MeOH was added dropwise. This was followed by a second gas evolution event. The reaction mixture was allowed to stir at rt for 1.5 h under a blanket of nitrogen. The reaction mixture was worked up by quenching with 1 M HCl until the pH reached between 1 and 2. The reaction mixture was then concentrated on a rotavap, and the resulting residue was dissolved in EtOAc and brine. The layers were separated, and the EtOAc layer was dried over MgSO_4_. EtOAc was evaporated, yielding the crude product. Yield: 111 mg (97%). ^1^H NMR (401 MHz, DMSO) δ (ppm) = 7.53 (s, 1H, CONH), 6.59–6.51 (m, 1H, CONH), 4.61 (t, *J* = 5.7 Hz, 1H, OH), 3.48–3.37 (m, 1H, CH), 3.25–3.18 (m, 1H, CH_2_), 3.17–3.07 (m, 2H, CH), 2.24–2.11 (m, 2H, CH_2_), 1.72–1.53 (m, 2H, CH_2_), 1.37 (s, 9H, BOC). ^13^C NMR (101 MHz, DMSO) δ (ppm) = 178.9, 155.6, 77.4, 64.0, 50.5, 37.8, 32.8, 28.2, 27.7.

***Synthesis of compound 3.4 (tert-butyl ((S)-1-oxo-3-((S)-2-oxopyrrolidin-3-yl)propan-2-yl) carbamate) – Scheme 4***

**Scheme 4. Synthesis of compound 3.4** **(8).**

Dess–Martin periodinane (392 mg, 0.93 mmol, 1.26 eq.) was dissolved in 3 mL dry DCM, containing tBuOH (78 µL, 60 mg, 0.81 mmol, 1.1 eq.) (8). Compound 3.3 (190 mg, 0.74 mmol, 1 eq.) was dissolved in 2 mL dry DCM and added to the solution of periodinane. The mixture was stirred at room temperature for 4 to 5 h (TLC control: EtOAc), then the reaction was quenched by the addition of sat. NaHCO_3_ (30 ml) and 1 M Na_2_S_2_O_3_ (30 mL) and intensive stirring for 15 min. The DCM layer was then separated, and the aqueous fraction was washed with DCM (3-4 × 15 ml). The combined organic layers were dried over MgSO_4_. The drying agent was removed by filtration and DCM was evaporated, giving the crude product. Yield: 135 mg (71%). ^1^H NMR (401 MHz, DMSO) δ (ppm) = 9.45 (s, 1H, CHO), 7.64 (s, 1H, CONH), 7.44 (d, *J* = 7.6 Hz, 1H, CONH), 3.95–3.84 (m, 1H, CH), 3.21–3.07 (m, 2H, CH_2_), 2.30–2.09 (m, 2H, CH_2_), 1.97 (d, *J* = 4.6 Hz, 1H, CH), 1.70–1.57 (m, 2H, CH_2_), 1.39 (s, 9H, BOC). ^13^C NMR (101 MHz, DMSO) δ (ppm) = 201.7, 178.3, 155.8, 78.5, 57.7, 37.4, 31.3, 29.5, 28.1, 27.3.

***Synthesis of compound 3.5*** ***(tert-butyl ((2S)-4-(benzylamino)-3-hydroxy-4-oxo-1-((S)-2-oxopyrrolidin-3-yl)butan-2-yl)carbamate) – Scheme 5***

**Scheme 5. Synthesis of compound 3.5 (9).**

Compound 3.4 (130 mg, 0.51 mmol, 1 eq.), benzyl isocyanide (73 µL, 70 mg, 0.6 mmol, 1.2 eq.) and pyridine (162 µL, 159 mg, 2 mmol, 4 eq.) were dissolved in 0.8 mL dry DCM (9). The mixture was stirred for 2 to 3 min under nitrogen and cooled to –15 °C (ice/methanol). Then, TFA (77 µL, 115 mg, 1 mmol, 2 eq.) was added dropwise over 1 h at –10 °C under nitrogen. The mixture was stirred for 2.5 h at –5 °C. The ice bath was then removed, and the mixture was left to come to ambient temperature. The reaction mixture was stirred at room temperature for 12 h (TLC control: EtOAc). When the aldehyde was depleted, DCM was evaporated, and the residue was dissolved in EtOAc and washed with sat. NaHCO_3_ (2×), 10% KHSO_4_ (2×) and brine (1×). The EtOAc layer was dried over MgSO_4_, the latter was filtered off, and EtOAc was removed by evaporation, yielding the crude product. The product was isolated as a mixture of alcohol and trifluoroacetyl ester, and then dissolved in 2 mL methanol. Next, 1.5 mL 4 M NaOH was added. Hydrolysis was allowed to proceed for 3 days, until the trifluoroacetyl ester disappeared (followed using HPLC). Methanol was evaporated, and the product was extracted with EtOAc (3 × 5 ml). The combined EtOAc layers were dried over MgSO_4_. After filtration and evaporation, the crude α-hydroxyamide was obtained. The product was dissolved in 4 mL acetic acid and purified by preparative RP HPLC with a gradient of 10 to 40% B. Yield: 26 mg (12.4%) as a mixture of diastereomers. LC–MS analysis: Two peaks at 6.26 min and 6.42 min with the monoisotopic molecular mass [M+H]^+^ 392.2, calculated 391.21 for C_20_H_29_N_3_O_5_.

***Synthesis of compound 3.6 (BOC-Abu-Tle-Leu-OH)***

Compound 3.6 was synthesized by solid phase synthesis on 2-CTC resin(10) (330 mg, substitution 1.6 mmol/g). Leucine was loaded as a Fmoc-Leu-OH (191 mg, 0.54 mmol, 1 eq.), dissolved in 5 ml dry DCM containing DIEA (365 µL, 277 mg, 2.15 mmol, 4 eq.). A basic solution of Fmoc-Leu-OH was loaded onto a 10 mL syringe that contained dry 2-CTC resin. The syringe with the resin and solution was shaken for 2 h at room temperature, then the liquid phase was discarded. The additional active sites on the resin were inactivated by washing the resin with DCM/MeOH/DIEA in a 17:2:1 ratio (3 × 3 mL for 1 min each), followed by washing with DCM (3 × 3 mL), DMF (2 × 3 mL) and DCM (5 × 3 mL). Then, the resin was dried under vacuum and lyophilized. Substitution of Fmoc-Leu-OH 0.143 mmol/g was measured by amino acid analysis method. The Fmoc-protecting group was removed by shaking the resin with 20% piperidine in DMF (2–3 mL) for 20 min. Then, the resin was thoroughly washed with DMF (3 × 3 mL), iPrOH (3 × 3 mL) and DMF (3 × 3 mL) till neutral pH detected by wet litmus. Subsequent amino acids were coupled by DIC/HOBt method in a 4:5:7 molar ratio of Fmoc-AA/HOBt/DIC in 1.5 mL DMF for 3 to 4 h. Then, the resin was washed by the above-mentioned scheme with DMF and iPrOH. The N-terminal amino acid was introduced with a BOC protecting group. The peptide was cleaved from the dried resin by shaking it with a 7:2:1 mixture of DCM/TFE/AcOH (6 mL) for 1 h, followed by washing with 2 mL of the same mixture for 10 min. Finally, the resin was washed with 4:1 DCM/TFE (2 × 3 mL). Combined solutions from the cleavage were evaporated, suspended in water, and lyophilized. LC–MS analysis: One peak at 4.07 min, corresponding to the monoisotopic molecular mass [M+Na]^+^ 451.750, calculated monoisotopic mass 429.28 for C_21_H_39_N_3_O_6_.

***Synthesis of compound 3.7 (BOC-Abu-Tle-Leu-3-amino-N-benzyl-2-hydroxy-4-((S)-2-oxopyrrolidin-3-yl)butanamide) – Scheme 6***

**Scheme 6. Synthesis of compound 3.7 (11, 12).**

Compound 3.5 (26.2 mg, 0.067 mmol, 1 eq.) was deprotected by dissolution in 0.6 mL acetonitrile, containing p-toluene sulfonic acid monohydrate (17 mg, 0.087 mmol, 1.3 eq.). The mixture was sonicated 6 × 5 min (HPLC control), then acetonitrile was removed on a rotavap. The tripeptide BOC-Abu-Tle-Leu-OH (3.6, 32 mg, 0.074 mmol, 1.1 eq.), PyBrOP (52 mg, 0.111 mol, 1.5 eq.) and HOBt (11.3 mg, 0.074 mmol, 1.1 eq.) was dissolved in 1 mL DCM. Deprotected compound 3.5 was dissolved in 0.5 mL DCM in a different flask, and the pH (wet litmus) was corrected by the addition of 7.5 µL DIEA to 8-9. The solution of the peptide and coupling agents was cooled to 0 °C, followed by the addition of an alkalized solution of deprotected compound 3.5 (11, 12). Then, DIEA (34 µL, 26 mg, 0.201 mmol) was added to the reaction mixture. The solution was left to stir for 5 min at 0 °C. The ice bath was then removed and the temperature was slowly increased to the ambient temperature. The reaction mixture was left to react for 12 h, and the pH was kept around 8. Then DCM was evaporated under nitrogen, and the residue was dissolved in 2 - 3 mL of EtOAc and extracted twice with sat. NaHCO_3_ (2×) and brine (1×). Then, the EtOAc solution was dried over MgSO_4_, the latter was filtered off and the EtOAc was removed by evaporation, yielding the crude product. LC–MS analysis: Three peaks at 4.22, 4.31, and 4.4 min with the monoisotopic molecular mass [M+H]^+^ 703.2, calculated 702.43 for C_36_H_58_N_6_O_8_.

***Synthesis of compound 3 (BOC-Abu-Tle-Leu-3-amino-N-benzyl-2-oxo-4-((S)-2-oxopyrrolidin-3-yl)butanamide) – Scheme 7***

**Scheme 7. Synthesis of compound 3 (9).**

Dess–Martin periodinane (55 mg, 0.13 mmol, 1.25 eq.) was dissolved in 1 mL dry DCM, containing tBuOH (11 µL, 8.5 mg, 0.114 mmol, 1.1 eq.). Compound 3.7 (73 mg, 0.104 mmol, 1 eq.) was dissolved in 0.5 mL dry DCM and added to the periodinane solution (5). The mixture was stirred at room temperature for 4 to 5 h (HPLC control), then quenched by the addition of sat. NaHCO_3_ (4.5 mL) and 1 M Na_2_S_2_O_3_ (4.5 mL) with intensive stirring for 15 min. The DCM layer was then separated, and the water fraction was washed with DCM (3-4 × 4 mL). The combined organic layers were dried over MgSO_4_. The drying agent was removed by filtration and DCM was evaporated, giving the crude product. The product was dissolved in 80% acetic acid and subjected to preparative HPLC (gradient 15–50% B). Yield: 8.1 mg as a mixture of diastereomers (11%). LC–MS analysis: Four peaks at 7.30, 7.49, 7.67, and 7.73 min with the monoisotopic molecular mass [M+H]^+^ 701.43, calculated 700.42 for C_36_H_56_N_6_O_8_. HR-ESI-MS analysis: Calculated 701.4238 for C_36_H_57_N_6_O_8_^+^, found [M+H]^+^ 701.4228, [M+Na]^+^ 723.4046. ^1^H NMR (401 MHz, DMSO) δ (ppm) = 9.26 (t, *J* = 6.4 Hz, 1H, COCONH), 8.47 (d, *J* = 7.8 Hz, 1H, CONH), 8.08 (d, *J* = 7.9 Hz, 1H, CONH), 7.64 (s, 1H, CONH), 7.41 (d, *J* = 9.4 Hz, 1H, CONH), 7.34–7.28 (m, 2H, aromatic), 7.27–7.23 (m, 2H, aromatic), 7.05 (d, *J* = 8.4 Hz, 1H, aromatic), 5.09 (t, *J* = 8.2 Hz, 1H, CH), 4.37–4.23 (m, 3H, CH + CH_2_), 3.90–3.79 (m, 1H, CH), 3.17 (t, *J* = 9.2 Hz, 1H, CH_2_), 3.06 (d, *J* = 6.8 Hz, 1H, CH_2_), 2.44–2.30 (m, 1H, CH), 2.20–2.10 (m, 1H, CH_2_), 1.95–1.86 (m, 1H, CH_2_), 1.70–1.63 (m, 1H, CH_2_), 1.63–1.54 (m, 2H, CH_2_), 1.53–1.44 (m, 1H, CH_2_), 1.37 (s, 9H, BOC), 1.33 (d, *J* = 5.9 Hz, 1H, CH), 0.88 (d, *J* = 3.5 Hz, 11H, CH_3_), 0.82 (t, *J* = 6.4 Hz, 6H, CH_3_). ^13^C NMR (101 MHz, DMSO) δ (ppm) = 196.5, 178.0, 172.0, 160.8, 138.5, 128.3, 127.3, 127.0, 78.1, 51.9, 34.8, 28.2, 27.1, 26.5, 26.3, 24.0, 22.7, 22.2, 21.9, 10.4.

***Fluorescent peptide substrate (Dabcyl-Asn-Arg-Abu-Orn-Leu-Gln-Ser-Gly-Asn-Ser-Arg-Lys-Edans)***

The side-chain-protected peptide Dabcyl-Asn(Trt)-Arg(Pbf)-Abu-Orn(Boc)-Leu-Gln(Trt)-Ser(tBu)-Lys(Boc)-Edans was synthesized using a standard Fmoc-chemistry solid-phase peptide synthetic protocol on 2-chlorotrityl chloride resin. The resin-bound peptide was cleaved with a mixture of acetic acid/2,2,2-trifluoroethanol/dichloromethane (2:2:6) for 2 h, evaporated, and dried *in vacuo*. Edans was then introduced using Edans acid sodium salt (1.5 eq.), PyBOP (1.0 eq.), and DIEA (3 eq. to protected peptide) in DMF for 2 h. After evaporating DMF, side-chain deprotection was achieved by incubation with a mixture of TFA/triisopropylsilane/water (95:2.5:2.5) for 1 h, followed by evaporation of solvents. The residue was purified using preparative RP-HPLC. LC/MS-ESI confirmed a molecular mass of [M+H] 1857.9.

***Analysis of peptide cleavage by LC–MS***

Samples were analyzed after 3 days of cleavage by both UV absorption at 210 nm and extracted ion current (EIC) of the intact peptides and target cleavage products (monitored masses: 724.04 for SGFRKM-NH_2_, 618.3 for TSAVLQ-OH, 1323.7 for TSAVLQSGFRKM-NH_2_, 560.3 for TSAVLA-OH, 1266.7 for TSAVLASGFRKM-NH_2_, 604.3 for TSAVLN-OH and 1309.7 for TSAVLNSGFRKM-NH_2_). Mature M^pro^ (110 nM) or preM^pro^ (4.8 μM) were used for cleavage of 200 μM substrates for 3 days, and 200 μM control peptides were used for comparison with cleavage data. Reactions proceeded in 20 mM Tris-HCl, pH 7.5, 200 mM NaCl, 1 mM EDTA, 1 mM DTT.

For LC–MS analysis of substrate cleavage, we employed an Agilent 6230 Accurate-Mass TOF LC/MS with Dual AJS electrospray ionization source (acquisition 2 GHz) and Agilent 1260 Infinity binary LC.

Peptide mixtures were separated using an Acquity UPLC BEH-C18 130 Å column (1.7 μm, 100 mm × 2.1-mm inner diameter; Waters) in a 10-min gradient from 2 to 100% mobile phase B (A, 0.1% aqueous FA; B, 0.1% FA in acetonitrile). The column temperature was 25 °C, and the flow rate was 0.3 mL/min.

The raw data are available at figshare.com, https://doi.org/10.6084/m9.figshare.26574340.v1.

**Supplementary refences**

1. Neises, B., and Steglich, W. (1978) Simple Method for the Esterification of Carboxylic Acids Angew Chem Int Ed Engl **17**, 522-524 10.1002/anie.197805221

2. Ede, N. J., and Bray, A. M. (1997) A simple linker for the attachment of aldehydes to the solid phase. Application to solid phase synthesis by the multipin™ method Tetrahedron Lett **38**, 7119-7122 10.1016/S0040-4039(97)01635-3

3. Sorg, G., Mengel, A., Jung, G., and Rademann, J. (2001) Oxidizing Polymers: A Polymer-Supported, Recyclable Hypervalent Iodine(V) Reagent for the Efficient Conversion of Alcohols, Carbonyl Compounds, and Unsaturated Carbamates in Solution Angew Chem Int Ed Engl **40**, 4395-4397 10.1002/1521-3773(20011203)40:23

4. Chen, M., and Yu, X. (2021) tert-Butyloxycarbonyl-protected amino acid ionic liquids and their application to dipeptide synthesis RSC Adv **11**, 27603-27606 10.1039/d1ra05597f

5. Vuong, W., Khan, M. B., Fischer, C., Arutyunova, E., Lamer, T., Shields, J. *et al.* (2020) Feline coronavirus drug inhibits the main protease of SARS-CoV-2 and blocks virus replication Nat Commun **11**, 4282 10.1038/s41467-020-18096-2

6. Tian, Q., Nayyar, N. K., Babu, S., Chen, L., Tao, J., Lee, S. *et al.* (2001) An efficient synthesis of a key intermediate for the preparation of the rhinovirus protease inhibitor AG7088 via asymmetric dianionic cyanomethylation of N-Boc-l-(+)-glutamic acid dimethyl ester Tetrahedron Letters **42**, 6807-6809 10.1016/S0040-4039(01)01416-2

7. Ghosh, A. K., Xi, K., Ratia, K., Santarsiero, B. D., Fu, W., Harcourt, B. H. *et al.* (2005) Design and synthesis of peptidomimetic severe acute respiratory syndrome chymotrypsin-like protease inhibitors J Med Chem **48**, 6767-6771 10.1021/jm050548m

8. Souček, M., Urban, J. (1995) An Efficient Method for Preparation of Optically Active N-Protected α-Amino Aldehydes from N-Protected α-Amino Alcohols Collect Czech Chem Commun **60**, 663-696 10.1135/cccc19950693

9. Semple, J. E., Owens, T. D., Nguyen, K., and Levy, O. E. (2000) New synthetic technology for efficient construction of alpha-hydroxy-beta-amino amides via the Passerini reaction Org Lett **2**, 2769-2772 10.1021/ol0061485

10. Román, T., Acosta, G., Cárdenas, C., de la Torre, B. G., Guzmán, F., and Albericio, F. (2023) Protocol for Facile Synthesis of Fmoc-N-Me-AA-OH Using 2-CTC Resin as Temporary and Reusable Protecting Group Methods Protoc **6**, 10.3390/mps6060110

11. Tichá, A., Stanchev, S., Vinothkumar, K. R., Mikles, D. C., Pachl, P., Began, J. *et al.* (2017) General and Modular Strategy for Designing Potent, Selective, and Pharmacologically Compliant Inhibitors of Rhomboid Proteases Cell Chem Biol **24**, 1523-1536.e1524 10.1016/j.chembiol.2017.09.007

12. Gandhi, S., Baker, R. P., Cho, S., Stanchev, S., Strisovsky, K., and Urban, S. (2020) Designed Parasite-Selective Rhomboid Inhibitors Block Invasion and Clear Blood-Stage Malaria Cell Chem Biol **27**, 1410-1424.e1416 10.1016/j.chembiol.2020.08.011
